# Differences in visual stimulation between reading and walking and implications for myopia development

**DOI:** 10.1167/jov.23.4.3

**Published:** 2023-04-04

**Authors:** Sabina Poudel, Hamed Rahimi-Nasrabadi, Jianzhong Jin, Sohrab Najafian, Jose-Manuel Alonso

**Affiliations:** 1 Department of Biological and Visual Sciences, SUNY College of Optometry, New York, NY, USA

**Keywords:** visual cortex, retina, thalamus, myopia, contrast, luminance, eye movements, freely moving

## Abstract

Visual input plays an important role in the development of myopia (nearsightedness), a visual disorder that blurs vision at far distances. The risk of myopia progression increases with the time spent reading and decreases with outdoor activity for reasons that remain poorly understood. To investigate the stimulus parameters driving this disorder, we compared the visual input to the retina of humans performing two tasks associated with different risks of myopia progression, reading and walking. Human subjects performed the two tasks while wearing glasses with cameras and sensors that recorded visual scenes and visuomotor activity. When compared with walking, reading black text in white background reduced spatiotemporal contrast in central vision and increased it in peripheral vision, leading to a pronounced reduction in the ratio of central/peripheral strength of visual stimulation. It also made the luminance distribution heavily skewed toward negative dark contrast in central vision and positive light contrast in peripheral vision, decreasing the central/peripheral stimulation ratio of ON visual pathways. It also decreased fixation distance, blink rate, pupil size, and head–eye coordination reflexes dominated by ON pathways. Taken together with previous work, these results support the hypothesis that reading drives myopia progression by understimulating ON visual pathways.

## Introduction

Retinal images are processed in the brain by ON and OFF visual pathways that signal different contrast polarities and have different spatiotemporal properties. High spatial frequencies (Jansen et al., 2019; Kremkow et al., 2014; Onat, Nortmann, Rekauzke, Konig, & Jancke, 2011) and large surfaces drive stronger response transients from ON than OFF pathways (Mazade, Jin, Pons, & Alonso, 2019; Mazade et al., 2022; Xing, Yeh, Gordon, & Shapley, 2014), whereas low retina illumination weakens the visual responses and receptive field surround of ON more than OFF pathways (Mazade et al., 2019; Rahimi-Nasrabadi et al., 2021). OFF pathways are also better represented in central vision (Jin et al., 2008; Masri, Grunert, & Martin, 2020; Williams et al., 2021) and have faster response dynamics than ON pathways (Jin, Wang, Lashgari, Swadlow, & Alonso, 2011; Komban et al., 2014; Mazade et al., 2019; Norcia, Yakovleva, Hung, & Goldberg, 2020; Rekauzke et al., 2016), and some of these ON–OFF temporal asymmetries are evolutionary preserved from invertebrates to humans (Komban et al., 2014; Leonhardt et al., 2016; Luo-Li, Mazade, Zaidi, Alonso, & Freeman, 2018).

ON and OFF visual pathways also respond differently to luminance contrast. The ON pathway saturates visual responses at lower contrasts than the OFF pathway (Archer, Alitto, & Usrey, 2021; Chichilnisky & Kalmar, 2002; Kremkow et al., 2014; Pons et al., 2017; Rahimi-Nasrabadi et al., 2021; Zaghloul, Boahen, & Demb, 2003), and this ON–OFF contrast difference expands the size of light more than dark stimuli. We call this size distortion neuronal blur because it blurs stimuli through neuronal responses instead of optics (Kremkow et al., 2014). The neuronal blur narrows the gap between closely spaced light stimuli, reducing the spatial resolution of ON more than OFF pathways and making small white letters more difficult to read than small black letters (Buchner & Baumgartner, 2007; Pons et al., 2017). This ON–OFF difference in spatial resolution is further amplified by light scatter and can be demonstrated with measurements of neuronal receptive field size (Chichilnisky & Kalmar, 2002; Kremkow et al., 2014), cortical retinotopy precision (Kremkow, Jin, Wang, & Alonso, 2016; K. S. Lee, Huang, & Fitzpatrick, 2016), human discrimination of gratings (Pons et al., 2017), and human visual sensitivity to Gaussian blur in image photography (Sato, Motoyoshi, & Sato, 2016). The neuronal blur also reduces the responses from ON visual pathways to low spatial frequencies because stimuli larger than the receptive field center activate more effectively the suppressive surround when expanded (Jansen et al., 2019; Kremkow et al., 2014; Onat et al., 2011). At the same time, the neuronal blur increases the visual responses from ON visual pathways to high spatial frequencies because stimuli smaller than the receptive field center increase their spatial summation when expanded (Pons et al., 2017).

The stimulus conditions that reduce visual stimulation of ON pathways also increase the risk of developing myopia, a visual disorder commonly known as nearsightedness that is becoming a world epidemic (Dolgin, 2015; Morgan & Jan, 2022). Optical blur, low light, and short viewing distance are all associated with an increase in myopia progression (Rose et al., 2008; Wallman & Winawer, 2004) and a weakening of visual responses from ON pathways (Jansen et al., 2019; Kremkow et al., 2014; Mazade et al., 2019; Mazade et al., 2022; Pons et al., 2019; Pons et al., 2017). The risk of developing myopia increases in tasks requiring vision at short distances such as reading (Ben-Simon et al., 2004; Pärssinen & Lyyra, 1993; Saw, Hong, Chia, Stone, & Tan, 2001; Zylbermann, Landau, & Berson, 1993), and as reading time increases with education level, the risk of developing myopia also increases (Shimizu et al., 2003; Sperduto, Seigel, Roberts, & Rowland, 1983; Wang, Klein, Klein, & Moss, 1994), sometimes within just one generation (Rozema et al., 2021; Wallman & Winawer, 2004). The risk of developing myopia also has a genetic component (Mutti, Mitchell, Moeschberger, Jones, & Zadnik, 2002; Mutti & Zadnik, 1995; Saw, Nieto, Katz, Schein, Levy, & Chew, 2001; Tkatchenko, Troilo, Benavente-Perez, & Tkatchenko, 2018) that is amplified by work at near distances (near work) and reduced by outdoor activity (Jones et al., 2007; Mutti et al., 2002; Pärssinen & Lyyra, 1993; Rose et al., 2008).

Differences in ON pathway activation can explain why reading and outdoor activity have opposite effects on myopia progression. Myopia can result from a deficit in retinal dopamine (Chakraborty et al., 2015; Iuvone, Tigges, Stone, Lambert, & Laties, 1991; Pardue et al., 2008), a neurotransmitter that is released by a single type of retinal amacrine cell driven by the ON pathway and stimulated by bright light (Zhang, Zhou, & McMahon, 2007). Spending time outdoors exposes the retina to bright light, which strongly increases the visual responses from ON pathways (Mazade et al., 2019; Mazade et al., 2022; Rahimi-Nasrabadi et al., 2021) and suppresses myopia progression (Ashby, Ohlendorf, & Schaeffel, 2009). Outdoor activity also stimulates ON pathways involved in reflexes of image–retina stabilization triggered by visual motion. These reflexes are extremely well preserved during evolution because they are crucial to remove motion blur and are seriously disrupted by ON-pathway deficits (Dryja et al., 2005; Emran et al., 2007; Kim, Joo, Han, & Woo, 2021; Kurata, Hosono, & Hotta, 2017; Winkelman et al., 2019). Therefore, an attractive hypothesis is that myopia progression is driven by a poor stimulation of ON visual pathways that disrupt the ON/OFF response balance (Aleman, Wang, & Schaeffel, 2018; Chakraborty et al., 2015; Crewther & Crewther, 2002; Crewther & Crewther, 2003; Iuvone et al., 1991; Pardue et al., 2008; Pons et al., 2019; Pons et al., 2017; Schwahn & Schaeffel, 1997; Smith, Fox, & Duncan, 1991). We test this hypothesis by quantitatively comparing the retinal stimulation of ON and OFF pathways in human subjects who performed two tasks associated with different risks of myopia, reading and walking. Reading has been intensively studied with psychophysical methods in the past, given its obvious importance in our society (Legge, Pelli, Rubin, & Schleske, 1985; Levi, Song, & Pelli, 2007; Pelli et al., 2007). Our results build on these previous studies and more recent work on myopia (Aleman et al., 2018) by demonstrating that reading causes a pronounced reduction in the visual stimulation of ON pathways.

## Methods

### Subjects

A total of eight subjects participated in the study, three with 20/20 vision (MB, SN, SP), three with myopia corrected to 20/20 vision (HRN corrected with prescription lenses from Tobii glasses, RM and JM corrected with contact lenses), one with uncorrected myopia (DL), and one with amblyopia and uncorrected myopia (JJ; see Table 1). We chose a small group of subjects with diverse optical conditions because, as the results demonstrate, the metrics of visual stimulation that we obtained were remarkably similar across diverse subjects but very different across tasks. Informed consent was taken from each subject prior to the experiment. The study was approved by the institutional review board at the State University of New York, College of Optometry, and followed the principles outlined in the Declaration of Helsinki.

**Table 1.** List of subjects.

Subject	Sex	Age (y)	Height (m)	Eye	Refractive error
1 (DL)	M	26	1.78	OD OS	−3.50/−5.00@180 (no refractive correction)−4.50/−5.00@005 (no refractive correction)
2 (MB)	F	28	1.50	ODOS	PlanoPlano
3 (SN)	M	28	1.75	ODOS	PlanoPlano
4 (HRN)	M	25	1.73	ODOS	−2.50 (corrected with Tobii lenses)−1.50 (corrected with Tobii lenses)
5 (RM)	M	31	1.88	ODOS	−2.75 (corrected with contact lenses)−2.75 (corrected with contact lenses)
6 (SP)	F	24	1.55	ODOS	PlanoPlano
7 (JM)	M	32	1.70	ODOS	−3.50 (corrected with contact lenses)−3.50 (corrected with contact lenses)
8 (JJ)	M	43	1.65	ODOS	−0.75/−3.50@80 (no refractive correction)amblyopia (LogMAR: 0.18, Snellen: 20/30)Plano

### Visual tasks

The subjects performed two visual tasks each lasting 5 min, reading and walking. All subjects performed the two tasks in a single day except JJ, who did it in 2 days. Before each task, eye position was calibrated with Tobii eye-tracking software by asking the subjects to fixate at the center of a circular target placed at a distance of 70 cm. In the reading task (Movie 1), all subjects were instructed to read a document on a computer screen as if they were at their offices or homes (without chinrest). The document was an article describing the epidemiology of myopia that did not include tables or figures but just text (page size: 25.5 × 26 cm, text font: 0.4 cm). To minimize eye-tracking noise, all subjects were asked to avoid touching the glasses while performing the tasks. In the walking task (Movie 2), the subjects were asked to walk indoors at their normal pace following a specific path. We chose to record the walking task indoors to maximize the quality of eye movement recordings, which decreases outdoors due to bright illumination (e.g., the large infrared content of sunlight interferes with the infrared lighting used for eye tracking). Walking indoors also allowed us to control more accurately the repeatability of the stimulus conditions across subjects. The subjects walked the path first with the recording turned off to become familiar with the task. After the practice run, they walked the same path again with the recording turned on while being followed by an instructor who reminded them where to go. The path had multiple corridors, stairs, and turning points. The subjects started at an office space within the 17th floor of the College of Optometry at the State University of New York (SUNY), walked through a long corridor, returned back through the same corridor, opened a door to reach a staircase that took them to the 15th floor, walked through multiple corridors on the 15th floor, went back to the staircase that took them to the 17th floor again, walked back and forth through the long corridor of the 17th floor, and then returned to the starting point (13 right turns and 9 left turns in total). The walls of the corridors had scientific posters that often attracted the attention of the subjects and were the target of occasional eye fixations. However, none of the subjects stopped at a poster and engaged in active reading (they were all instructed to walk without interruption through the designated path). During the reading task, the maximum luminance was 90 cd/m^2^ (e.g., white page or white text) and the minimum 2 cd/m^2^ (e.g., black text or black page). During the walking task, the lights, walls, floors, and ceilings of the corridors had the following luminance ranges: 1,600–5,000 cd/m^2^, 90–145 cd/m^2^, 70–90 cd/m^2^, and 40–65 cd/m^2^. The wall and floor of the staircases had the following luminance ranges: 10–30 cd/m^2^ and 2–5 cd/m^2^. The differences in retinal illumination across stimulus conditions depend on both pupil size and stimulus luminance. To document these differences, we measured the pupil size and retinal illumination of two subjects (Subjects 4 and 6) under three different conditions: reading white text on a black background, reading black text on a white background, and looking at a bright sky through an office window. The average pupil diameter of these two subjects was 4.24 ± 0.29 mm when reading on a black background (4.03 and 4.44 mm), 3.70 ± 0.66 mm when reading on a white background (3.23 and 4.16 mm), and 2.09 ± 0.03 mm when fixating at a bright sky through an office window (2.11 and 2.07 mm). The luminance was 2 cd/m^2^ for the black background, 90 cd/m^2^ for the white background, and 9,500 cd/m^2^ for the blue sky. The average retinal illuminance of the two subjects was 28.22 trolands for the black background, 965.94 trolands for the white background, and 32,484.11 trolands for the bright sky. Therefore, when compared with looking at a black monitor screen, the retinal illuminance increases by more than one order of magnitude when looking at a white monitor screen and by more than three orders of magnitude when looking at a bright sky.

### Data acquisition

While performing the two tasks, the subjects wore a set of Tobii Pro Glasses 2 that monitored their visual behavior. The Tobii Pro Glasses 2 have two main units, the glasses and a recording box. The glasses were similar in size and weight to any pair of commercial glasses. They were equipped with a frontal camera to record the visual scene, as well as four infrared cameras (two per eye) and 12 infrared lights (six per eye) to record the eye movements. The glasses also had two inertial motion sensors (one accelerometer and one gyroscope) to record the linear acceleration and angular velocity of the head movements along three axes (x: pitch, y: yaw, z: roll). Pitch, yaw, and roll describe, respectively, the head rotation within the x-axis (looking up or down), y-axis (looking left or right), and z-axis (tilting the head left or right while looking at front). The scene camera captured videos with a resolution of 1,920 × 1,080 pixels at a rate of 25 frames per second (position [0 0] at top left corner and [1,920 1,080] at bottom right). The infrared cameras captured eye videos of 240 × 960 pixels at a rate of 50 frames per second (position [0 0] at top left corner and [240 × 960] at bottom right). The inertial motion sensors sampled linear acceleration and angular velocity at a rate of 100 Hz. The eye tracker had an accuracy of ∼ 0.8 degrees and a range of ± 40 degrees. During each recording session, the glasses were connected through a high-definition multimedia interface (HDMI) cable to a recording box attached to the subject waist.

### Data analysis

The recording box stored the scene movies, eye movies, time stamps, and visuomotor data in a removable secure digital (SD) card. The movies were stored in mp4 files and the time stamps/visuomotor data in JavaScript Object Notation (JSON) files. Custom MATLAB software was used to extract the following information from these files: time stamps of the inertial motion sensors sampled at 100 Hz (ts), images from the scene camera sampled at 25 Hz (a time stamp every 16 scene images, vts), images from the eye cameras sampled at 50 Hz (a time stamp every 25 eye images, evts), two-dimensional (2D) gaze position in scene-camera space (gp) ranging from [0 0] at the top left corner of the scene to [1 1] at the bottom right corner, three-dimensional (3D) gaze position (gp3) ranging from a value of [0 0 0] millimeters at the center of the Tobii glasses (where the scene camera is located) to the viewing distance, pupil diameter (pd) from each eye measured in millimeters, linear acceleration measured by the accelerometer (ac) in meters per squared seconds, and angular velocity measured by the gyroscope (gy) in degrees per second. The 3D gaze was converted from millimeters to degrees of visual angle by calculating the arctangents of the ratio between horizontal image distance and visual depth distance (for horizontal gaze position) and between vertical image distance and visual depth distance (for vertical gaze position). The 3D gaze movements were classified into four different categories using an algorithm developed by Pekkanen and Lappi (2017): fixation, smooth pursuit, saccades, and postsaccadic oscillation (see classification of eye movements below).

The scene camera had a viewing angle of 82 × 52 degrees and captured images of 1,920 × 1,080 pixels. Therefore, the 82 degrees of the horizontal image axis were sampled with 1,920 pixels (23 pixels per degree) while the 52 degrees of the vertical image axis were sampled with 1,080 pixels (21 pixels per degree). All scene images were converted to 8-bit grayscale using the “rgb2gray” MATLAB function and stacked into a luminance matrix (*L*(*s*, *t*)), where *s* is the pixel spatial position and *t* is time. To analyze stable retinal projections of the visual scene, we selected periods of time when the eye was fixating or in smooth pursuit. Anatomically, the human fovea is an area of retinal depression at the center of the macula with a diameter of 1.5 mm that covers approximately 5 degrees of visual field. Therefore, to analyze the images projected in the fovea, we selected a circular portion of the scene with a diameter of 5 degrees centered at the gaze point. Immediately surrounding the fovea, there is a retinal region with an external diameter of 2.5 mm called the parafovea that extends approximately 8 degrees of visual field. We defined peripheral retina as the region between the parafovea and the far periphery (8 to 60 degrees) and the peripheral scene as a ring centered on the fixation point with inner and outer diameters of 8 and 60 degrees, respectively. The lower and upper halves of this peripheral scene were analyzed separately to identify possible differences between vision in lower and upper visual fields. Because the number of image pixels is larger in the visual periphery than the fovea (radius: 52 vs. 5 degrees), we randomly selected 1,000 pixels from each retinal region (s: 1 to 1,000) when comparing measurements between retinal regions or tasks. Sampling the same number of pixels at the fovea and visual periphery is important for statistical comparisons and to simulate the reduction in visual sampling with retinal eccentricity. Adding Gaussian blur to the peripheral images caused only modest changes in spatiotemporal contrast and luminance skewness (Gaussian blur with a standard deviation of 23 pixels reduced spatial-temporal contrast by 2–3% and spatial-temporal luminance skewness by 0.01–0.1). Therefore, all analyses of visual periphery were done with unprocessed images (no added Gaussian blur) undersampled to match the number of pixels at the fovea (random sample of 1,000 pixels).

In each scene, we measured the following spatiotemporal parameters: spatial contrast, temporal contrast, spatial skewness of the luminance distribution, and temporal skewness of the luminance distribution. Contrast was defined as the root mean square (RMS) of the difference between the intensity of each pixel *L*(*s*,*t*), which ranged from 0 to 1, and the average pixel intensity (µ, RMS contrast, Equation 1). To measure spatial contrast (*C_S_*) at each time (*t*), the intensity of each pixel was subtracted from the average pixel intensity across space (*µ_S_* (*t*)). Then, the sum squared difference for all pixels across space was divided by the number of sampled pixels (N_S_ = 1,000). To measure temporal contrast (*C_T_*) at each pixel spatial location (*s*), the intensity of each pixel was subtracted from the average pixel intensity across time (*µ_T_* (*s*)). Then, the sum squared difference for all pixels across time was divided by the number of sampled pixels (*N_T_* = number of images at times of eye fixation or smooth pursuit).

(1) $$\begin{array}{c} \begin{array}{c} C_{S}\left(t\right)=\sqrt{\frac{1}{N_{S}-1}\sum_{s}{\left(L\left(s,t\right)-\mu_{S}\left(t\right)\right)}^{2}\;}\\ C_{T}\left(s\right)=\sqrt{\;\frac{1}{N_{T}-1}\sum_{t}{\left(L\left(s,t\right)-\mu_{T}\left(s\right)\right)}^{2}}\; \end{array}\; \end{array}$$

The spatial and temporal skewness of the luminance distribution (*SK_S_*(*t*), *SK_T_*(*s*)) were calculated as the mean difference between each pixel luminance (*L*(*s,t*)) and their mean (*µ*), normalized by the RMS contrast (*C*) and elevated to the power of 3 (Equation 2). To measure spatial skewness (*SK_S_*) at each time (*t*), the luminance of each pixel was subtracted from the average pixel luminance across space (*µ_S_*(*t*)) and the difference normalized by the spatial contrast (*C_s_*(*t*)). Then, the normalized difference was elevated to the power of 3 and averaged across all pixel locations (*N_S_* = 1,000). To measure temporal skewness (*SK_T_*) at each spatial location (*s*), the luminance of each pixel was subtracted from the average pixel luminance across time (*µ_T_*(*s*)) and the difference normalized by the temporal contrast (*C_T_*(*s*)). Then, the normalized difference was elevated to the power of 3 and averaged by the number of sampled pixels across time (*N_T_* = number of images at times of eye fixation or smooth pursuit). A negative skewness indicates that there is more variation in luminance contrast on the negative side of the luminance distribution (dark pixels), a positive value indicates that there is more variation on the positive side (light pixels), and a value close to zero indicates similar variation in both sides (equal variation in luminance contrast for light and dark pixels).

(2) $$\begin{array}{c} \begin{array}{c} SK_{S}\left(t\right)=\frac{1}{N_{S}}\sum_{s}{\left(\frac{L\left(s,t\right)\;-\;\mu_{S}\left(t\right)}{C_{S}\left(t\right)}\right)}^{3}\\ SK_{T}\left(s\right)=\frac{1}{N_{T}}\sum_{t}{\left(\frac{L\left(s,t\right)\;-\;\mu_{T}\left(s\right)}{C_{T}\left(s\right)}\right)}^{3} \end{array}\; \end{array}$$

We also calculated the mean pixel intensity of all images across time. The average pixel intensity was similar at the fovea and visual periphery during walking but higher at the fovea than visual periphery during reading black text on a white background. However, we did not perform systematic comparisons of absolute pixel intensity across tasks because the Tobii camera automatically adjusts pixel intensity to avoid image luminance saturation and does not currently provide any way to control or monitor these automatic adjustments. The relation between luminance and pixel intensity of the Tobii scene camera was roughly linear. The linear correlation coefficient calculated by exposing the camera to a sequence of calibrated light flashes with variable luminance was 0.95. We also measured the spatial frequency spectrum of the scenes by averaging the spectrums of all images captured during each of the tasks.

We calculated the following visuomotor parameters: fixation depth, fixation duration, right and left pupil diameter, horizontal and vertical eye position, horizontal and vertical eye velocity, blink rate, total eye tracking time, head velocity/acceleration in different axes, and total head motion. The fixation depth was imported from the Tobii JSON file only for eye movements classified as fixations. The fixation duration was calculated as the time duration of eye movements that were classified as fixations or smooth pursuit. The pupil diameter from each eye (in millimeters) and the eye position (in millimeters) were imported from the Tobii JSON file. The eye position was converted from millimeters to degrees as explained above. The eye velocity was calculated as the ratio between saccade amplitude and duration (only for eye movements classified as saccades). The saccade amplitude was computed as the square root of the summed squared amplitudes of horizontal and vertical eye positions. The blink rate was calculated with a custom algorithm applied to the Tobii movies of the eyes (see description of blink detection algorithm below). The total eye-tracking time was imported directly from the Tobii JSON file and quantifies the time during which the eye tracking was not interrupted by movement or illumination artifacts. The total eye-tracking time was very similar across subjects and tasks. The mean, standard deviation, and range of eye-tracking time across subjects were 246.6 ± 2.2 [243.7 to 249.2] seconds for reading and 244.7 ± 4.9 [237.6 to 249.2] seconds for walking. Head acceleration and velocity along the three spatial axes (pitch, yaw, and roll) were extracted directly from the Tobii JSON file. We also calculated the average eye movement following a head turn to the right, left, up, or down. For this analysis, we generated time stamps marking the times when the head velocity was above a specified threshold (2.5 times the median velocity for each head direction, one time stamp every 50 ms). We then averaged the eye movements centered at each head movement time stamp.

### Classification of eye movements

We classified eye movements in fixations, smooth pursuit, saccades, and postsaccadic oscillation using a naive segmented linear regression (NSLR) algorithm developed by Pekkanen and Lappi (2017). The NSLR algorithm (https://gitlab.com/nslr/nslr) takes as input the horizontal and vertical gaze angles from Tobii glasses. Then, it converts each of the gaze recordings in a sequence of linear segments by iteratively minimizing the difference between the original recording and the reconstructed segments. It starts with a single segment that reconstructs the mean slope of the entire recording and then decreases the difference between original and reconstructed recordings as more segments are incorporated in the reconstruction. The NSLR uses a prior exponential distribution of segment durations to avoid incorporating very short segments. After the segment reconstruction is finished, the algorithm uses a four-state hidden Markov Model (HMM) to classify eye movements into four possible types (states): fixation, saccade, smooth pursuit, and postsaccadic oscillations. The HMM uses Gaussian distributions of segment velocity and intersegment angles for each type of eye movement as prior probability distributions to perform the classification. It also incorporates a trained probability of HMM state transitions to maximize classification accuracy (e.g., postsaccadic oscillations cannot precede saccadic movements).

### Blink detection

We trained a convolutional neural network (CNN) to classify images acquired with one of the Tobii eye cameras (left eye) as eyes open or eyes closed. We selected 1,000 eye images for the reading task (161 images of eyes closed and 839 images of eyes open from five subjects) and 1,060 images for the walking task (205 images of eyes closed and 855 images of eyes open from eight subjects). We trained the network with 242 eye images from the reading task and 308 from the walking task. For each task, half of the training images had eyes closed and the other half eyes opened. The training data set was further augmented by randomly rotating the images within −45 to 45 degrees, translating them in both axes by −1 to 1 pixel, and randomly inverting them over the y-axis. To speed up the processing time, each image was resized to 36 × 36 pixels in a grayscale format. The network was trained with a stochastic gradient descent algorithm using a batch size of 128 images and 500 epochs. At the end of training, we validated the performance of the network with both the training images (training data set) and the images not used for training (validation data set made of 758 images for reading and 752 for walking).

The network had three convolutional layers with 16, 32, and 64 filters. Each filter had 3 × 3 pixels with values that were stochastically modified during the network training (range: −1 to 1). The first network layer convolved each image with 16 filters (16 convolution arrays per image). It then performed a batch image normalization in each convolution array by subtracting the mean and then dividing by the standard deviation of a randomly selected subset of 128 image convolutions (batch size). The normalized convolution arrays were then passed through a rectified linear unit (ReLu) activation function that had a linear positive slope and a threshold stochastically modified during the training. After the batch normalization and rectification, the network used maxpooling to reduce the data dimensionality by converting the 36 × 36 convolution arrays to 18 × 18 arrays. The maxpooling was performed by assigning the maximum of each 2 × 2 pixels from the convolution array to one of the pixels of the 18 × 18 new array. The second network layer convolved the 18 × 18 array with a set of 32 filters and then performed batch normalization, ReLU activation, and maxpooling as in the first layer. The maxpooling in the second layer assigned the maximum of each 2 × 2 pixels from the first-layer array to each pixel of a new 9 × 9 array. The third network layer convolved the 9 × 9 arrays with a set of 64 filters and fed the values of all arrays to a fully connected layer with two neurons that use softmax activation functions. The activation of each neuron in this last layer reflects the probability that the eye in a given image is open or closed (the summed activation probability of both neurons is always 1). The performance of each epoch was validated with a subset of eye images (758 for reading, 752 walking), and the epoch that achieved the best performance was used to select the CNN for blink detection. The accuracy of the network estimated with the training and validation image data sets was higher than 95% (reading: 97% with training data set and 99% with validation data set; walking: 98% with training data set and 96% with validation data set). Blink duration was calculated by counting the number of consecutive eye images classified by the algorithm as eye closed and multiplying this number by the image duration. Blinking frequency was calculated by counting the number of blinks within time intervals of 15 s.

### Measurements of horizontal image flow

We measured the average horizontal motion direction of the scene by processing pairs of consecutive images with an algorithm developed by Gunnar Farnebäck (2003). The algorithm approximates the direction and magnitude of image displacement through an iterative process that uses five image resolution scales, with 10 iterations per scale. It starts with the scale of highest resolution and refines the measurements by decreasing the resolution two times every 10 iterations. The vector direction of the image displacement is calculated by fitting a quadratic function to windows of 5 × 5 pixels from two consecutive images and then extracting the magnitude and direction of the image displacement from the fits. The quadratic function is defined as *f*(*x*) = *x^T^ Ax* + *b^T^ x* + *c*, where *x* is the vector describing the relative pixel position in the image, and *A*, *b*, and *c* are variables modified during the fitting procedure (*A* is a symmetric matrix, *b* is a vector, and *c* is a scalar). The error in the fitting procedure is weighted with a Gaussian function centered at the 5 × 5-pixel matrix, and the fitting is iterated across different window positions within a region of 25 × 25 pixels to smooth the estimates of image displacement.

After the vector displacements were calculated, we measured the vector average across all image pixels within the horizontal axis of the scene, which we call average horizontal flow. We then selected the peaks in horizontal flow larger than 20% of the maximum across subjects, which occurred when the subject made walking turns, which often triggered an optokinetic reflex. We then selected segments of horizontal eye movements centered on these horizontal flow peaks (±1 s around the peak, 100 data points sampled at 50 Hz per segment) that were not seriously disrupted by blinks (at least 60 data points) and were obtained when the subject was walking (we did not include the last 10 last seconds of the recordings at the end of the walking task). The selected recording segments of horizontal eye movements were then processed with a fast Fourier transform to extract the average amplitude and frequency of maximum amplitude between 2 and 6 Hz.

### Statistical analysis

We assessed statistical significance with two-tailed Wilcoxon tests when comparing parameter distributions between reading and walking tasks. We fit the average distributions across subjects for each specific parameter with Gaussian (Equation 3) or Alpha functions (Equation 4) described as follows:

(3) $$\begin{array}{c} y=b+a\;e^{-\frac{{\left(x-\mu \right)}^{2}}{2\;\sigma^{2}}}\; \end{array}$$

where *b* is the baseline, *a* is the amplitude, µ is the mean, and σ is the standard deviation of the Gaussian function.

(4) $$\begin{array}{c} y=max\left[0,\;b+\;d\left(\frac{\left(x-a\right)}{r}\;e^{\frac{1-\left(x-a\right)}{r}}\right)\right]\; \end{array}$$

where *b* is the baseline, *d* is the parameter controlling the decay phase, *r* is the parameter controlling the raising phase, and *a* is the origin of the raising phase from the Alpha function.

## Results

**Figure 1.**
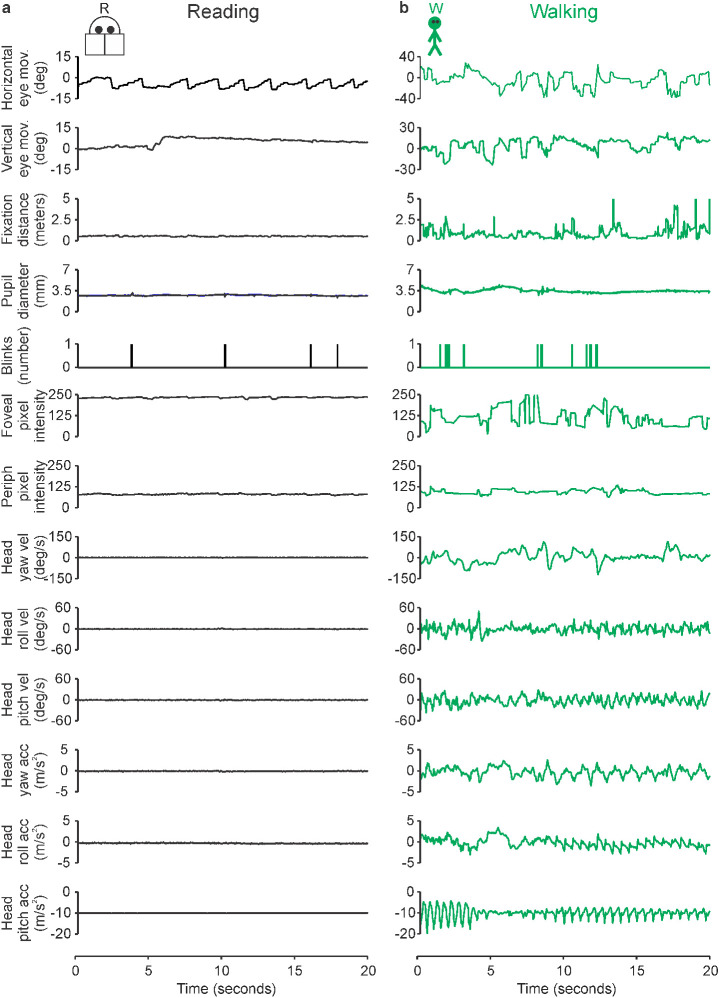
Example recordings from two visual tasks, reading and walking. (a) Different measurements of visual behavior in one subject during the reading task. From top to bottom, horizontal eye movements, vertical eye movements, fixation distance, pupil diameter, blink raster, foveal pixel intensity, peripheral pixel intensity, head yaw velocity, head roll velocity, head pitch velocity, head yaw acceleration, head roll acceleration, and head pitch acceleration. (b) Same for walking task.

We asked human subjects to perform two tasks associated with different risk of myopia progression, reading (high risk) and walking (low risk). In the reading task, the subjects sat down at an office desk and read a text on a computer monitor. In the walking task, the same subjects walked through a specific path of corridors within the SUNY Optometry building. The subjects wore glasses with cameras and sensors that allowed us to quantify the main differences between the two tasks in the image sequences projected in the retinas (Figure 1). As expected, reading generated repetitive eye movements that oscillated between the two ends of each line of text and between the top and bottom of the page (Figure 1a, first and second panels from the top). The eye movements during reading were accompanied by limited changes in fixation distance, blink rate, pupil diameter, image luminance, and head velocity/acceleration (Figure 1a). When compared with reading, walking generated a much more variable pattern of eye movements (Figure 1b, first and second panels from the top, notice scale difference with Figure 1a) that were accompanied by more pronounced variations in nearly all visual parameters that we measured (Figures 1a, b). The differences between the two tasks were quantified by comparing the distributions of multiple parameters extracted from the visual scene and visuomotor activity. Below, we provide a systematic comparison of a large number of parameters measured, which aims to be as complete as possible and unbiased by current knowledge on eye growth (Baird et al., 2020; Wallman & Winawer, 2004). For simplicity, we use the term *reading* to describe reading black text on a white background, which is the most common reading format, but we also report additional measurements for reading white text on a black background.

### Reading black text on a white background reduces the stimulation of ON visual pathways in central vision

Reading and walking projected very different sequences of images in the retina. During reading, the images were centered on a pattern of black letters that remained relatively constant over time. Conversely, during walking, the image pattern was continuously changing and dominated by a complex combination of exploratory movements from eyes, head, head–eye coordination reflexes, body oscillations (e.g., footsteps), and scene transitions. We started the quantification of these pronounced differences in visual input by measuring the distributions of spatial and temporal RMS contrast in the scene. The RMS contrast increases as the number of light and dark pixels becomes equal and activates ON and OFF pathways more effectively.

**Figure 2.**
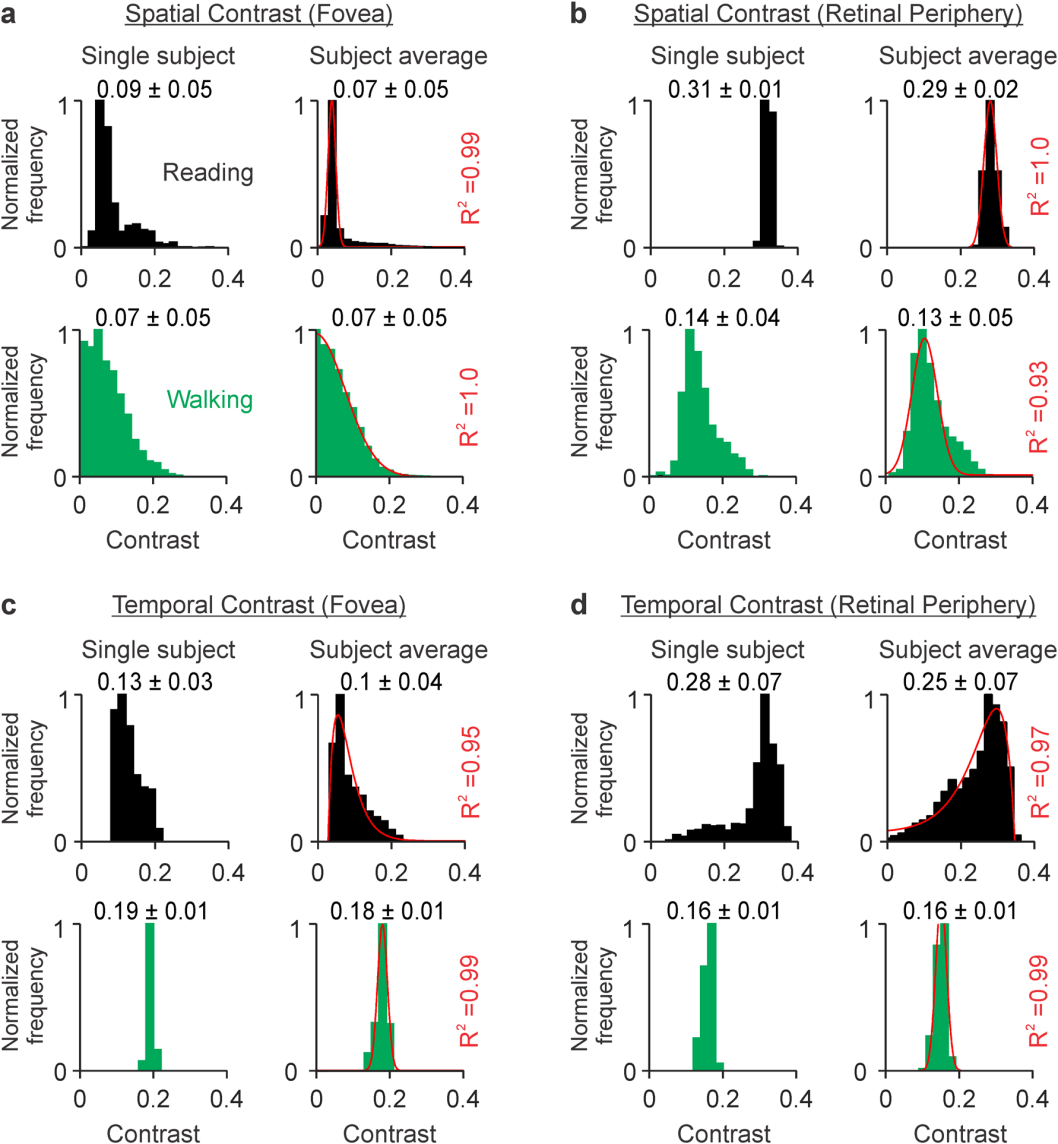
Fovea–periphery differences in spatiotemporal contrast are more pronounced when reading black text on a white background than when walking. (a) RMS spatial contrast measured at the fovea of an example subject (left) and the average across subjects (right) during reading (black, top row) and walking (green, bottom row). The numbers on the top of each histogram are means ± standard deviations of spatial contrasts, which range from 0 to 1. The red lines are Gaussian fits (goodness of fit on the right). The [means, standard deviations] of the Gaussian fits are [0.038, 0.01] for reading and [0.003, 0.081] for walking. (b) Same as a, for visual periphery. The [means, standard deviations] of the Gaussian fits are [0.275, 0.017] for reading and [0.104, 0.034] for walking. (c) Same as a, for temporal contrast. The red lines are an Alpha fit for reading and a Gaussian fit for walking. The [mean, standard deviation] of the Gaussian fit are [0.177, 0.013]. (d) Same as b, for temporal contrast. The red lines are an Alpha fit for reading and a Gaussian fit for walking. The [mean, standard deviation] of the Gaussian fit are [0.145, 0.015].

At the fovea, the distributions of spatial contrast could be accurately fit with Gaussian functions in both tasks (Figure 2a), but the shapes of the functions were very different. The most frequent spatial contrast at the fovea (peak of the Gaussian) was one order of magnitude higher for reading than walking (0.038 for reading vs. 0.003 for walking), whereas the diversity of contrasts (Gaussian width) was nearly one order of magnitude higher for walking than reading (0.081 for walking vs. 0.01 for reading). Surprisingly, despite these pronounced differences in the shape of the contrast distributions, the means and standard deviations for reading and walking were nearly identical (0.07 ± 0.05 for reading and 0.07 ± 0.05 for walking). The means were similar because the contrast distribution was much broader for walking than reading, which made the mean for walking higher than the peak of the Gaussian. The standard deviations were similar because the contrast distribution for reading had a long tail caused by fixations near the edge of the page, which had high contrast. Unlike the fovea, the mean contrast at the visual periphery was twice as high for reading than walking (Figure 2b, mean: 0.29 ± 0.02 for reading vs. 0.13 ± 0.05 for walking) because the high-contrast edge of the page fell most of the time at the visual periphery (note that the fovea and periphery cover very different image areas but are both sampled with 1,000 pixels; see Methods for more details).

The distributions of temporal contrast were also very different between the two tasks (Figures 2c, d). Whereas the distributions for reading were asymmetric and best fit with Alpha functions, the distributions for walking were symmetric, much narrower, and best fit with Gaussian functions. The mean temporal contrast was lower for reading than walking at the fovea (0.1 ± 0.04 for reading vs. 0.18 ± 0.01 for walking), but the difference was reversed at the visual periphery (0.25 ± 0.07 for reading vs. 0.16 ± 0.01 for walking). During reading, the spatiotemporal contrasts were different between fovea and periphery because the border of the white page (projected in the peripheral retina) had higher contrast than the middle of the page (projected at the fovea). Unlike reading, walking projected images at the fovea and periphery that were frequently interchanged, making their contrast distributions more similar (e.g., an image at the fovea falls in the periphery when the fovea moves to a different target). It is important to emphasize that all these measurements (and those reported below) were very similar across individual subjects. Although each subject reads and walks differently, the metrics that we are reporting have remarkably small individual variability (see measurements for each subject in Supplementary Figure S1). Based on the differences in spatial and temporal contrast that we report, we conclude that walking drives the fovea with higher temporal contrast than reading, but reading drives the visual periphery with higher spatiotemporal contrast than walking.

**Figure 3.**
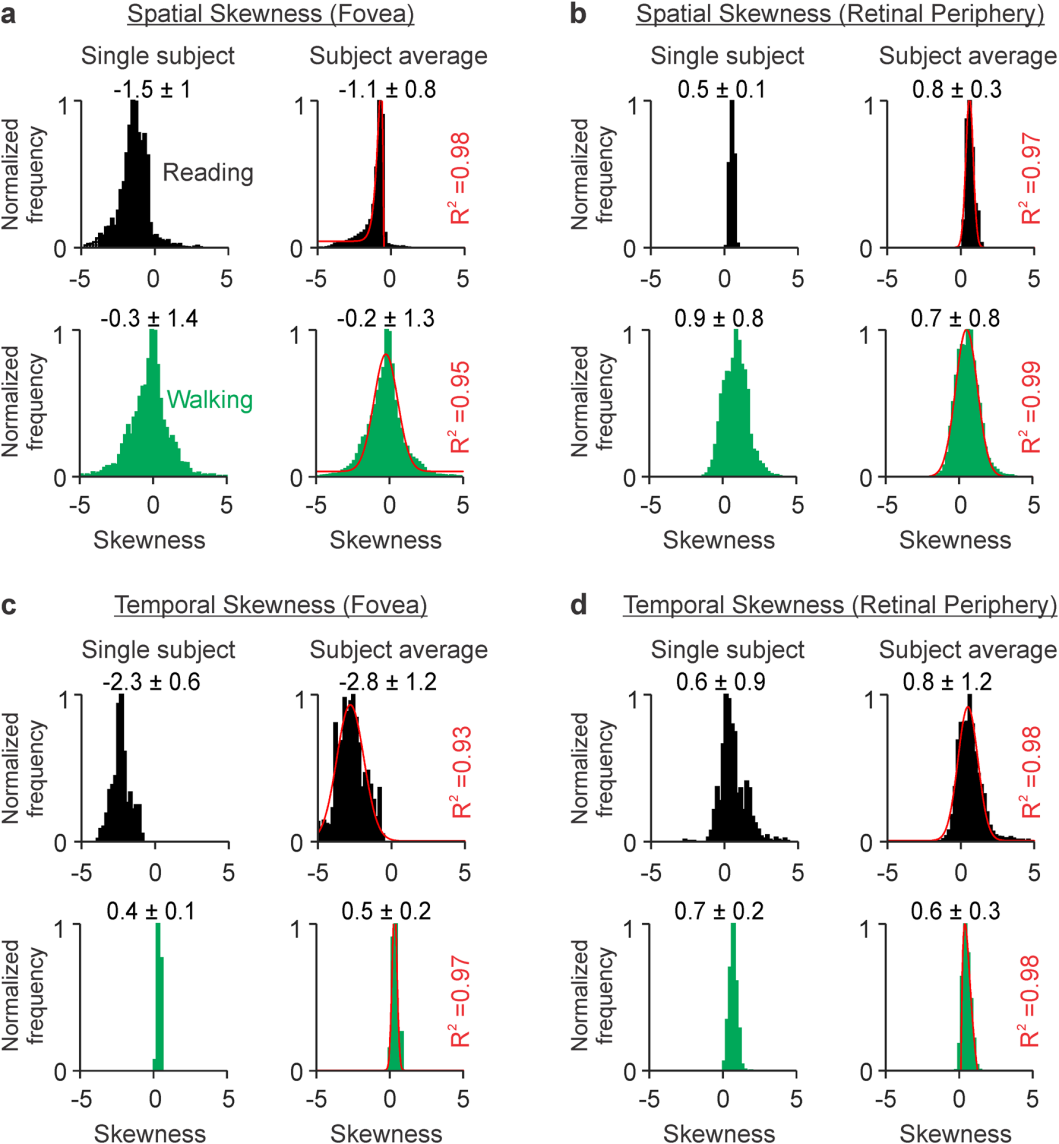
Reading black text on a white background causes a pronounced bias toward dark contrasts in visual stimulation. (a) Spatial skewness measured at the fovea of an example subject (left) and the average across subjects (right) during reading (black, top row) and walking (green, bottom row). The format is the same as in Figure 2. The [raising, decaying] phases of the Alpha fit for reading are [−0.2, 1.0]. The [mean, standard deviation] of the Gaussian fit for walking are [−0.1, 0.9]. (b) Same as a, for visual periphery. The [means, standard deviations] of the Gaussian fits are [0.6, 0.3] for reading and [0.5, 0.7] for walking. (c) Same as a, for temporal skewness. The [means, standard deviations] of the Gaussian fits are [−2.8, 0.9] for reading and [0.3, 0.2] for walking. (d) Same as b, for temporal skewness. The [mean, standard deviation] of the Gaussian fit for reading are [−0.5, 0.7]. The [raising, decaying] phases of the Alpha fit for walking are [0.3, 1.3].

Reading and walking also generated luminance distributions with very different skewness, a measure that is negative when images are biased toward dark contrasts, positive when biased toward light contrasts, and zero when light and dark contrasts are balanced (see Methods for details). Reading black letters on a white background generated skewness distributions heavily dominated by dark contrasts at the fovea (Figures 3a, c, black histograms, average: −1.1 ± 0.8 for spatial and −2.8 ± 1.2 for temporal skewness) and more modestly dominated by light contrasts at the visual periphery (Figures 3b, d, black histograms, average: 0.8 ± 0.3 for spatial and 0.8 ± 1.2 for temporal skewness). By comparison, walking generated a more balanced distribution of light and dark contrasts at the fovea (Figure 3a, green histograms, average: −0.2 ± 1.3) and a modest dominance of light contrasts at the visual periphery (Figure 3b, green histograms, average: 0.7 ± 0.8). Consequently, the fovea–periphery difference in absolute skewness was significantly lower for walking than reading (−0.83 ± 1.48 vs. −1.81 ± 0.9 for spatial skewness; −0.13 ± 0.31 vs. −3.68 ± −1.80 for temporal skewness, *p* < 0.00001 for both, Wilcoxon tests). Walking also generated a much narrower distribution of temporal skewness than reading (Figures 3c, d, green histograms). Based on these skewness differences, we conclude that walking drives a more balanced stimulation of ON and OFF visual pathways than reading, in both fovea and peripheral retina (see measurements for each subject in Supplementary Figure S2).

Most books have printed black text on a white background, but white text on a black background is becoming increasingly more available in digital devices. White text on a black background has been shown to increase choroidal thickness in the human eye of emmetropes and myopes (Aleman et al., 2018; Swiatczak & Schaeffel, 2022), a process that is associated with myopia suppression in animal models (Wildsoet & Wallman, 1995). Patients with low vision also prefer reading white text on a black background for reasons that remain unclear (Legge, Rubin, Pelli, & Schleske, 1985). Therefore, to make our study more complete, we obtained additional measurements for reading white text on a black background in two of our subjects. In both subjects, white text had two to four times higher spatial and temporal contrast than black text at the fovea (Supplementary Figure S3a, b) because the light scatter expanded the size of white letters on the black background while shrinking the size of black letters on the white background. The contrast difference between black and white text increased with optical blur, a reduction in the camera spatial resolution, and an increase in the contrast–response saturation of ON visual pathways (Kremkow et al., 2014; Pons et al., 2017; Rahimi-Nasrabadi et al., 2021). However, the difference was also present (although reduced) in measurements with a high-resolution camera that had limited optical blur and a linear contrast–response function (Nikon D850 camera of ∼19 million pixels). Our measurements may explain why patients with low vision prefer reading white text on a black background, as their optical deficits and retinal cell loss should make the contrast higher for white than black text. White text on a black background also made the luminance distribution biased toward light contrasts and reduced the differences in contrast and skewness between fovea and periphery (see Supplementary Figure S3c, d). Therefore, we conclude that reading white text on a black background provides a more balanced stimulation of ON and OFF visual pathways than reading black text on a white background.

**Figure 4.**
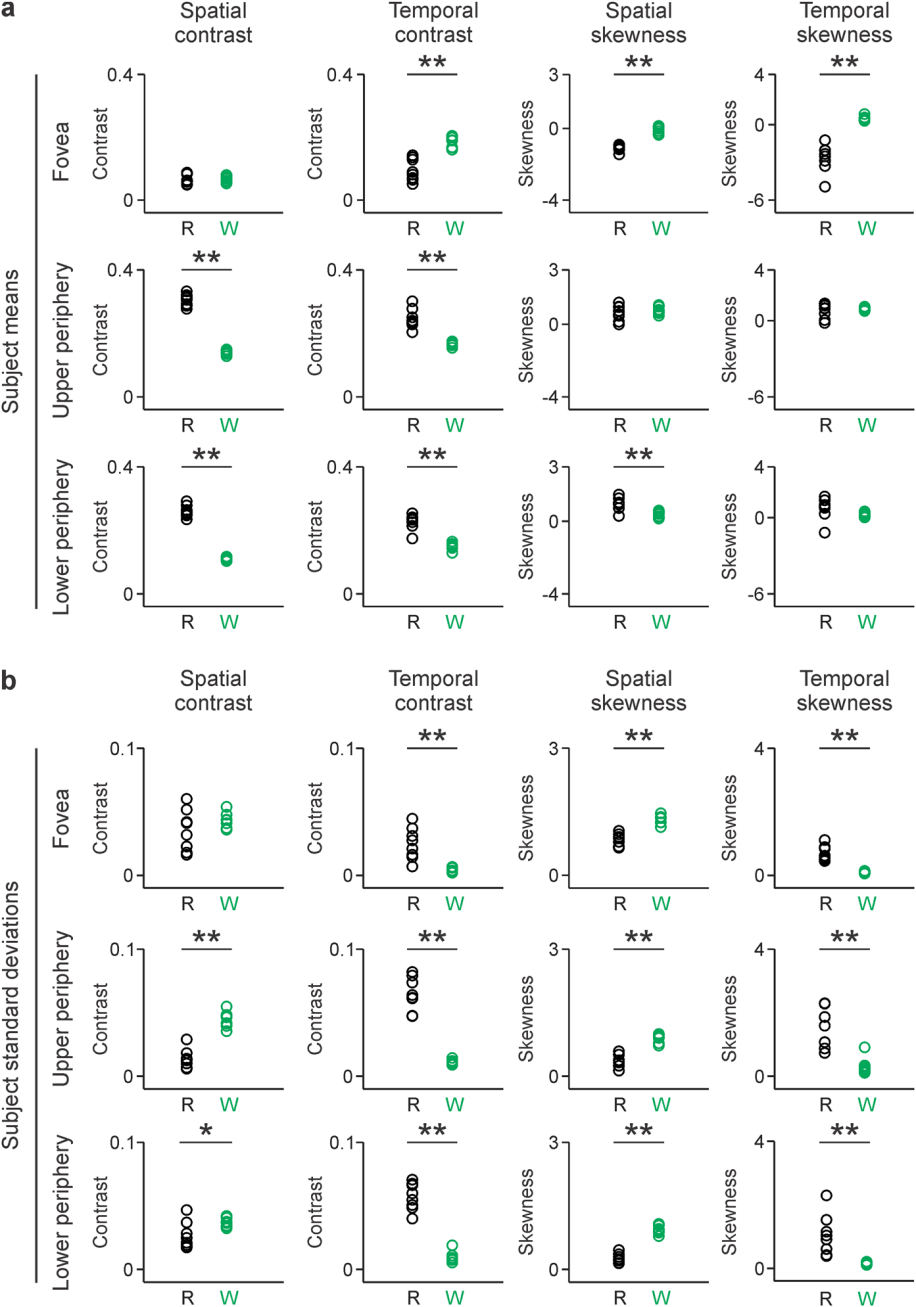
Reading and walking generate images with different spatiotemporal contrast and luminance skewness. (a) Means of different spatiotemporal measurements. From left to right, spatial contrast, temporal contrast, spatial skewness, and temporal skewness, measured at different retinal regions in the eight subjects (fovea, upper periphery, and lower periphery). Color code for reading and walking as in previous figures. (b) Same as a for standard deviations of the measurements. *p < 0.05. **p < 0.01.

Similar conclusions could be reached if we compare the average values of contrast and skewness from individual subjects instead of the average subject distribution (Figure 4). The statistical power of this comparison is limited to eight values, one per subject. However, this comparison is important because it illustrates very clearly the limited variability across subjects and the more pronounced differences across tasks. Consistently with the previous analyses, walking stimulated the fovea with higher temporal contrast than reading (Figure 4a, top), but reading stimulated the visual periphery with higher spatiotemporal contrast than walking (Figure 4a, middle and bottom). Walking also stimulated the fovea with much more balanced dark/light contrast than reading, as indicated by the smaller skewness values. In fact, during walking, the dark/light balance was nearly perfect in all subjects, and the values of spatiotemporal skewness were all very close to zero (Figure 4a, right).

When compared with reading, walking also caused more variation (larger standard deviation) in spatial skewness and much less variation in temporal contrast and temporal skewness in both fovea and visual periphery (Figure 4b). In fact, the variation in temporal skewness during walking was nearly zero for all subjects at the fovea and lower periphery (Figure 4b, right panels). This is a surprising finding given that walking generates more variability than reading in nearly all other visual parameters (see below). We conclude that walking drives the retina with a pattern of temporal stimulation that is well balanced for dark/light contrast and very similar across subjects.

It is important to emphasize that the differences between reading and walking that we report are not restricted to the fovea but affect a large area of central vision. We quantify in detail central–periphery differences at the two extremes of visual resolution, but the reading page covers a retinal area larger than the fovea. Therefore, to better understand the size of central vision affected by reading, we measured the gradient of spatiotemporal contrast and skewness stimulating retinal areas of different sizes. We calculated the average spatial contrast, temporal contrast, spatial skewness, and temporal skewness within circular portions of the scene of different sizes centered at the point of fixation (5, 10, 15, 20, 25, 30, and 60 degrees in diameter). As expected from our previous measurements, increasing the visual area from 5 to 60 degrees made the average spatiotemporal contrast higher and the spatiotemporal skewness closer to 0 (i.e., it increased the ON–OFF stimulation balance). The averages reached 50% of the maximum–minimum range at 17.3 degrees for spatial contrast, 15.5 degrees for temporal contrast, 32.8 degrees for spatial skewness, and 33.2 degrees for temporal skewness. Therefore, we conclude that reading black text on a page of ∼25 cm^2^ at a distance of ∼0.5 m (see below) drives the 15–30 degrees of central vision with half the contrast and half the ON–OFF balance than peripheral vision.

### Reading causes a pronounced reduction of visuomotor activity

**Figure 5.**
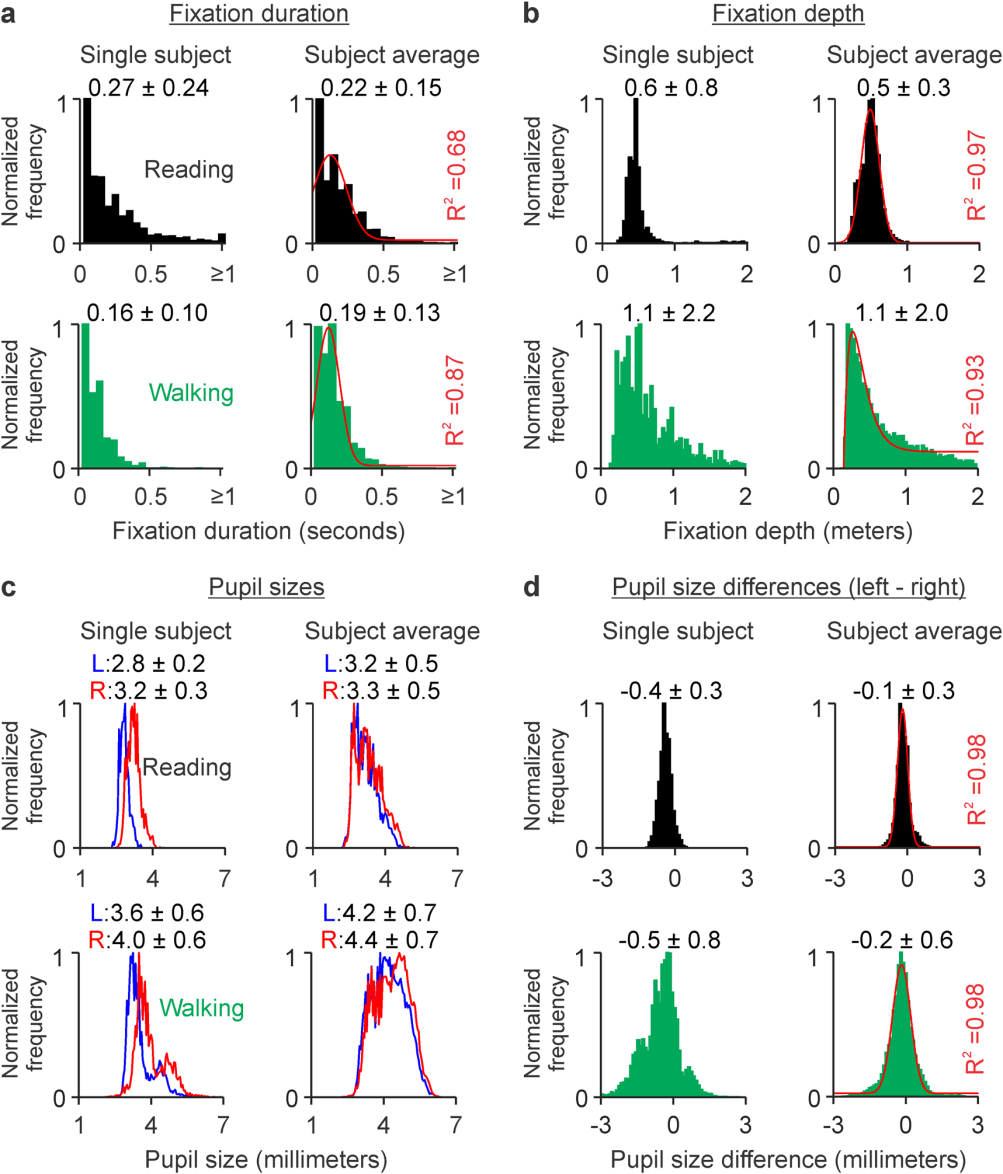
When compared with walking, reading increases fixation duration but decreases fixation distance and pupil size. (a) Fixation duration measured in one example subject (left) and the average across subjects (right), during reading (black, top row) and walking (green, bottom row). The format is the same as in Figure 2. The [means, standard deviations] of the Gaussian fits are [0.1, 0.1] for reading and [0.1, 0.1] for walking. (b) Same as a, for fixation distance. The [mean, standard deviation] of the Gaussian fit for reading are [0.5, 0.1]. The [rising, decaying] phases of the Alpha fit for walking are [0.1, 0.8]. (c) Line plots of left (blue) and right (red) pupil sizes. (d) Same as a, for pupil size differences (left–right pupil sizes). The [means, standard deviations] of the Gaussian fits are [−0.2, 0.2] for reading and [−0.2, 0.4] for walking.

A major difference between reading and walking is body movement. Consistently, nearly all measurements of visuomotor activity were larger and more variable during walking than reading (Movie 3). The only exception was the average fixation duration (Figure 5a), which was 30 ms longer and 20 ms more variable during reading than walking (Figure 5a, reading/walking: 0.22 ± 0.15/0.19 ± 0.13 s, *p* < 0.00001, Wilcoxon test). The fixation distance during reading was nearly constant at about 0.5 m, very similar across subjects (Figure 5b, black histograms, 0.5 ± 0.3), and normally distributed. Conversely, the fixation distance was two times larger, at least five times more variable (Figure 5b, green histograms, 1.1 ± 2.0), and better fit with Alpha functions during walking. The average pupil size was also 1 mm larger during walking than reading, as would be expected from the larger average fixation distance (Figure 5c). The variations in pupil size and pupil size differences between the eyes were also larger during walking than reading (Figures 5c, d) consistently with the more frequent eccentric gazing during walking that occasionally made one eye closer to fixation than the other (see measurements for each subject in Supplementary Figure S4). When compared with black text, reading white text on a black background increased the pupil size by 0.3–0.8 mm (black text vs. white text: 3.23 ± 0.25 vs. 4.03 ± 0.1 mm, *p* = 0.0002 for Subject 4, 4.16 ± 0.17 vs. 4.44 ± 0.17 mm, *p* = 0.0028 for Subject 6, Wilcoxon tests) and decreased fixation duration by 39–57 ms (black text vs. white text: 227.9 ± 8.7 vs. 171.0 ± 7.6 ms, *p* = 0.0079 for Subject 4, 239.2 ± 18.1 vs. 200.0 ± 9.7 ms, *p* = 0.0079 for Subject 6, Wilcoxon tests).

The variations in eye position and saccade velocity were also larger during walking than reading (see Supplementary Figure S5), but there were some biases that are worth reporting. For example, the subject with amblyopia made more fixations on the left visual field in front of his fellow eye than on the right visual field in front of his amblyopic eye (Supplementary Figure S5a, green histograms, Subject 8). Also, during reading, the eyes and heads of all subjects had a higher average velocity toward the left than the right side of the page, as expected from a left-to-right reading pattern (Supplementary Figure S5c, black histograms, average: −25 ± 202 deg/s; Supplementary Figure S6a, average: −1.6 ± 3.6 deg/s). Eye movements, head velocity (Supplementary Figure S6a, b), and acceleration (Supplementary Figure S6c, d) were all more variable and had larger amplitude during walking than reading, and the average distributions could be fit well with Gaussian functions. With the exception of the bias for fast head velocities to the left during reading, most distributions of head movement had a mean close to zero in the two tasks, indicating that head velocities and accelerations are similar across all directions (Supplementary Figure S6).

**Figure 6.**
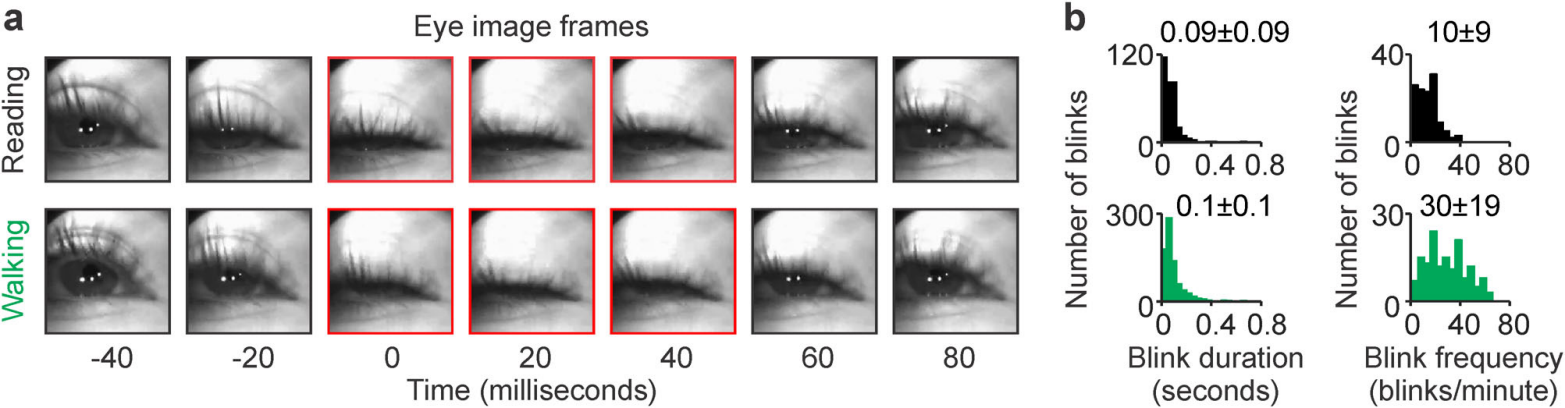
Reading reduces blink duration and frequency. (a) Eye images from an example subject illustrating an eye blink while reading (top row) and walking (bottom row). (b) Histograms of blink duration (left) and frequency (right) measured across subjects during reading (black, top row) and walking (green, bottom row).

Another important difference in visuomotor activity between reading and walking was in the dynamics of eye blinks. The average blink duration was 13 ms longer and 19 ms more variable during walking than reading (Figures 6a, b, walking vs. reading: 98.23 ± 113.31 vs. 85.27 ± 94.02, *p* = 4.85 × 10^−5^, Wilcoxon test). Also, the average blink rate was three times higher and two times more variable during walking than reading (Figure 6b, walking vs. reading: 30 ± 19 vs. 10 ± 9, *p* = 1.27 × 10^−25^, Wilcoxon test; see measurements for each subject in Supplementary Figure S7). Luminance transients (including those generated by blinks) are very effective at driving visual responses from ON pathways (Jin, Wang, Lashgari, et al., 2011; Komban et al., 2014; Mazade et al., 2019; Mazade et al., 2022; Xing et al., 2014). Moreover, the response strength from ON pathways increases with both the luminance intensity and the duration of the dark period preceding the luminance transient (Mazade et al., 2019). Therefore, by reducing the frequency of blink-driven luminance transients and the duration of the dark period preceding the transient (i.e., duration of eye closed), reading should activate ON visual pathways less effectively than walking.

**Figure 7.**
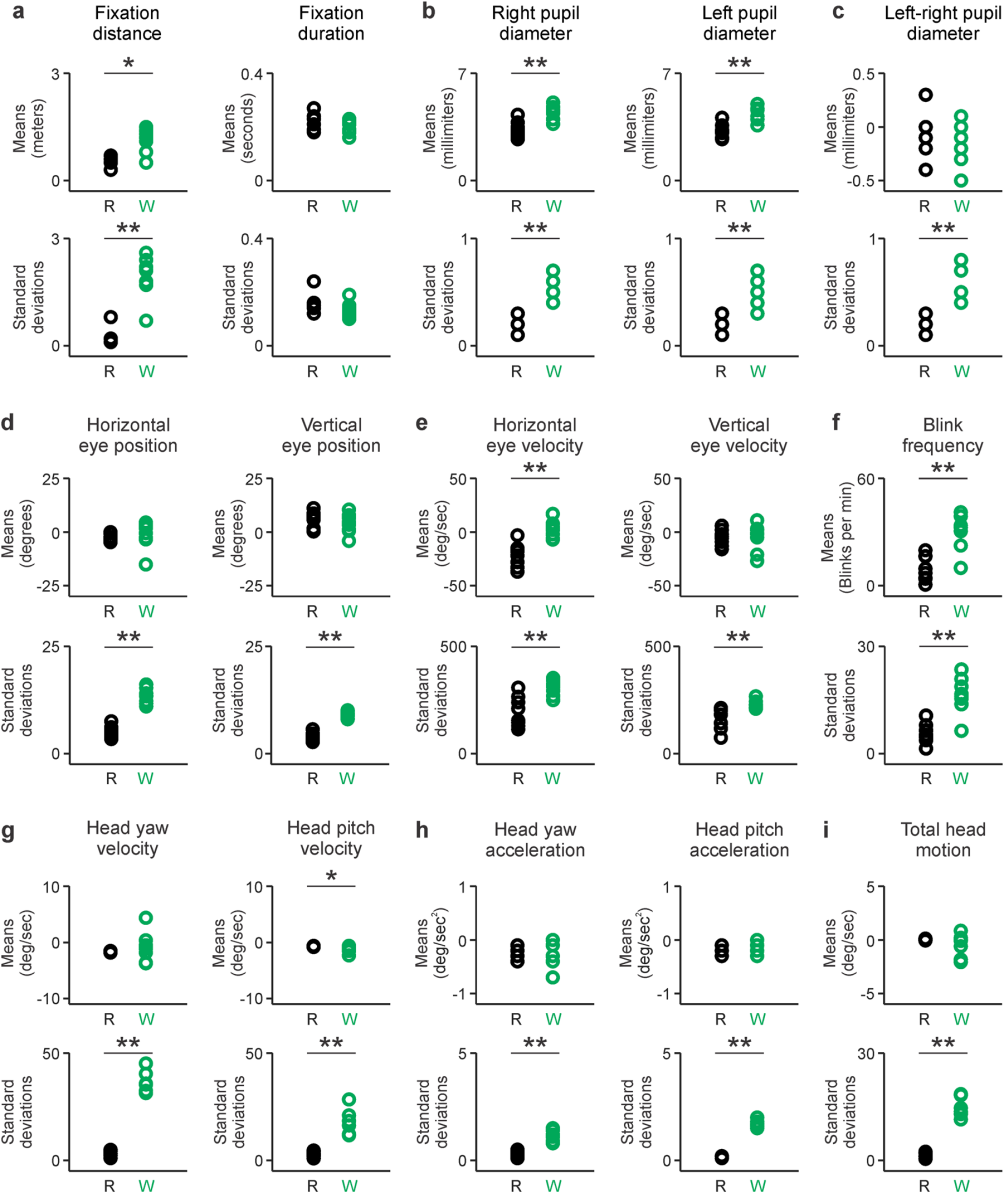
Reading drives less variation in visuomotor activity than walking. (a) Means (top row) and standard deviations (bottom row) of fixation distance (left) and fixation duration (right). Same format for the other figure panels. (b) Right and left pupil diameters. (c) Left–right difference in pupil diameter. (d) Horizontal and vertical eye positions. (e) Horizontal and vertical eye velocities. (f) Blink frequency. (g) Head yaw and pitch velocities. (h) Head yaw and pitch accelerations. (i) Total head motion (average of head velocity across the three axes). *p < 0.05. **p < 0.01.

The measurements of visuomotor activity described above could be also replicated by comparing the means across subjects. When compared with reading, walking generated greater variations in fixation distance (Figure 7a), larger and more variable pupil diameters (Figure 7b), more variable pupil–diameter differences between the two eyes (Figure 7c), and more variability in horizontal eye position, vertical eye position, horizontal eye velocity, vertical eye velocity (Figures 7d, e, bottom), blink frequency (Figure 7f), head velocity, and acceleration (Figures 7g–i). Therefore, we conclude that reading causes a pronounced reduction of visuomotor activity when compared with walking.

### Reading decreases the activation of head–eye image stabilization reflexes

The reduction of head movements during reading also decreased the activation of head–eye coordination reflexes involved in image retinal stabilization (Schweigart, Mergner, Evdokimidis, Morand, & Becker, 1997). Head–eye coordination reflexes are evolutionary well preserved and are needed to minimize motion blur and maintain visual acuity during visual navigation. Across the animal kingdom, from fish to mammals, the neuronal circuits involved in image retinal stabilization (e.g., optokinetic reflexes) are heavily dominated by ON visual pathways (Emran et al., 2007; Sugita, Miura, Araki, Furukawa, & Kawano, 2013). Therefore, a decrease in the activation of head–eye image stabilization movements decreases the visual stimulation of these ON pathways. Reading and walking generated different oscillation frequencies in head velocity (Supplementary Figure S8). During reading, the dominant frequency in head pitch velocity was around 0.5 Hz or lower (Supplementary Figure S8a, c). Instead, during walking, the footsteps dominated head pitch velocity with a frequency around 2 Hz (Supplementary Figures 8b, d). Therefore, when compared with reading, walking increased the average frequency of head pitch velocity by nearly five times (Supplementary Figure S8c, d) and, by doing so, increased the need for fast head–eye coordinated movements to stabilize the retinal image.

**Figure 8.**
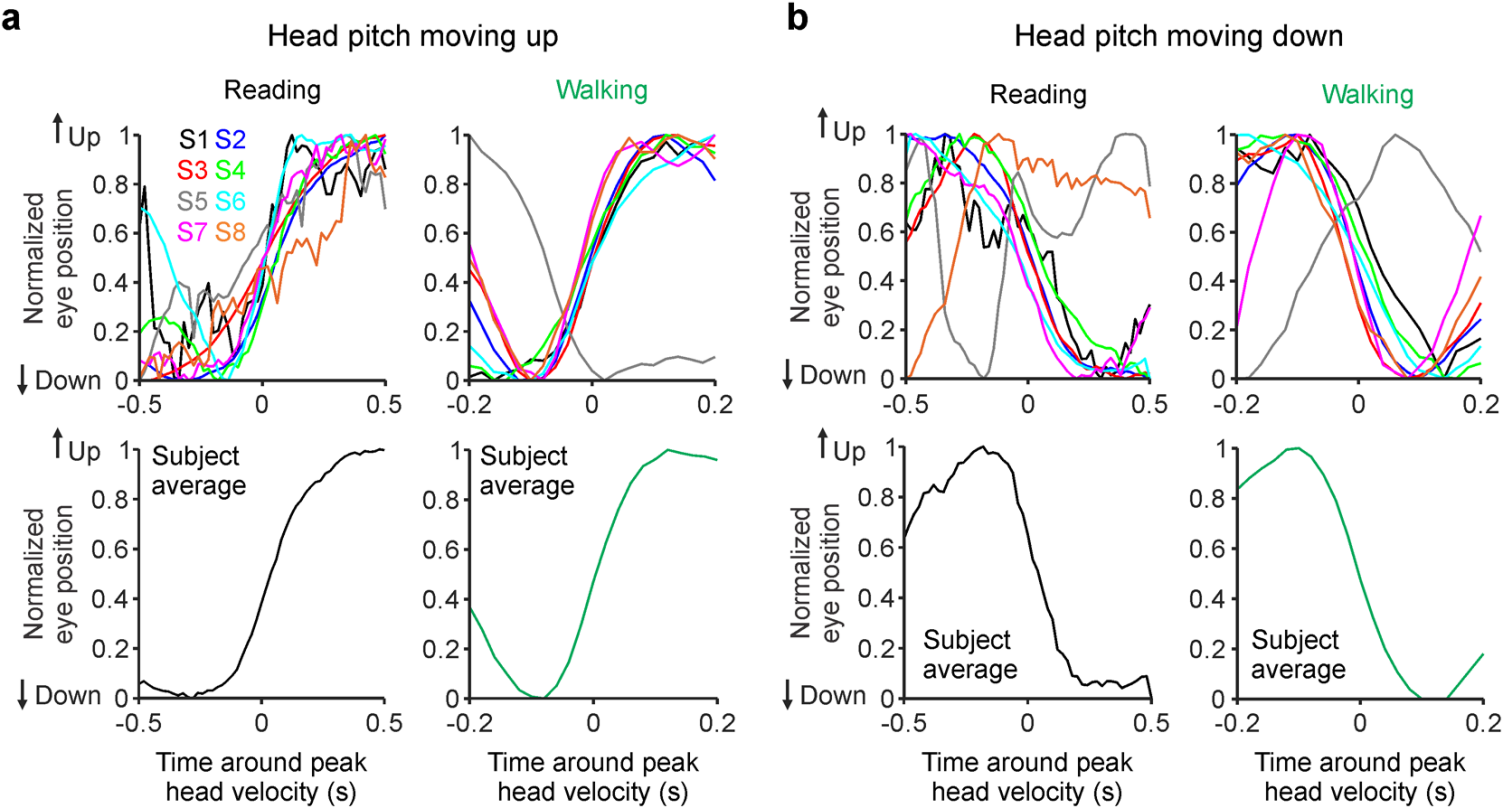
Head-triggered eye movements are slower during reading than walking. (a) Normalized eye position centered at the point of maximum head velocity for reading (top left) and walking (top right), shown for each individual subject (top) and the subject average (bottom). The scale in the x-axis is two times larger for reading than walking because the head-triggered eye movements were two times slower. (b) Same as a, for head pitch moving down. The time course was similar across most subjects but slower than the average for one subject with corrected myopia (S5, gray lines) and a subject with amblyopia (S8, orange line).

To measure the dynamics of head–eye coordination reflexes, we calculated the average eye movement associated with the fastest head pitch oscillations in each subject (see Methods for details). The head–eye coordinated movements identified by these analyses were remarkably similar across subjects. Upward movements of the head were closely associated with upward movements of the eye during both reading and walking. Although the movement amplitude was variable across subjects (Supplementary Figure S9), the time course of the head–eye coordinated movements was remarkably similar across individuals and tasks (Figures 8a, b). The main difference was in the duration of the head–eye coordination reflex, which was about two times faster during walking than reading (Figures 8a, b, note scale difference in x-axis). Based on these results, we conclude that walking doubles the speed of head–eye coordination reflexes needed to stabilize the retinal image.

**Figure 9.**
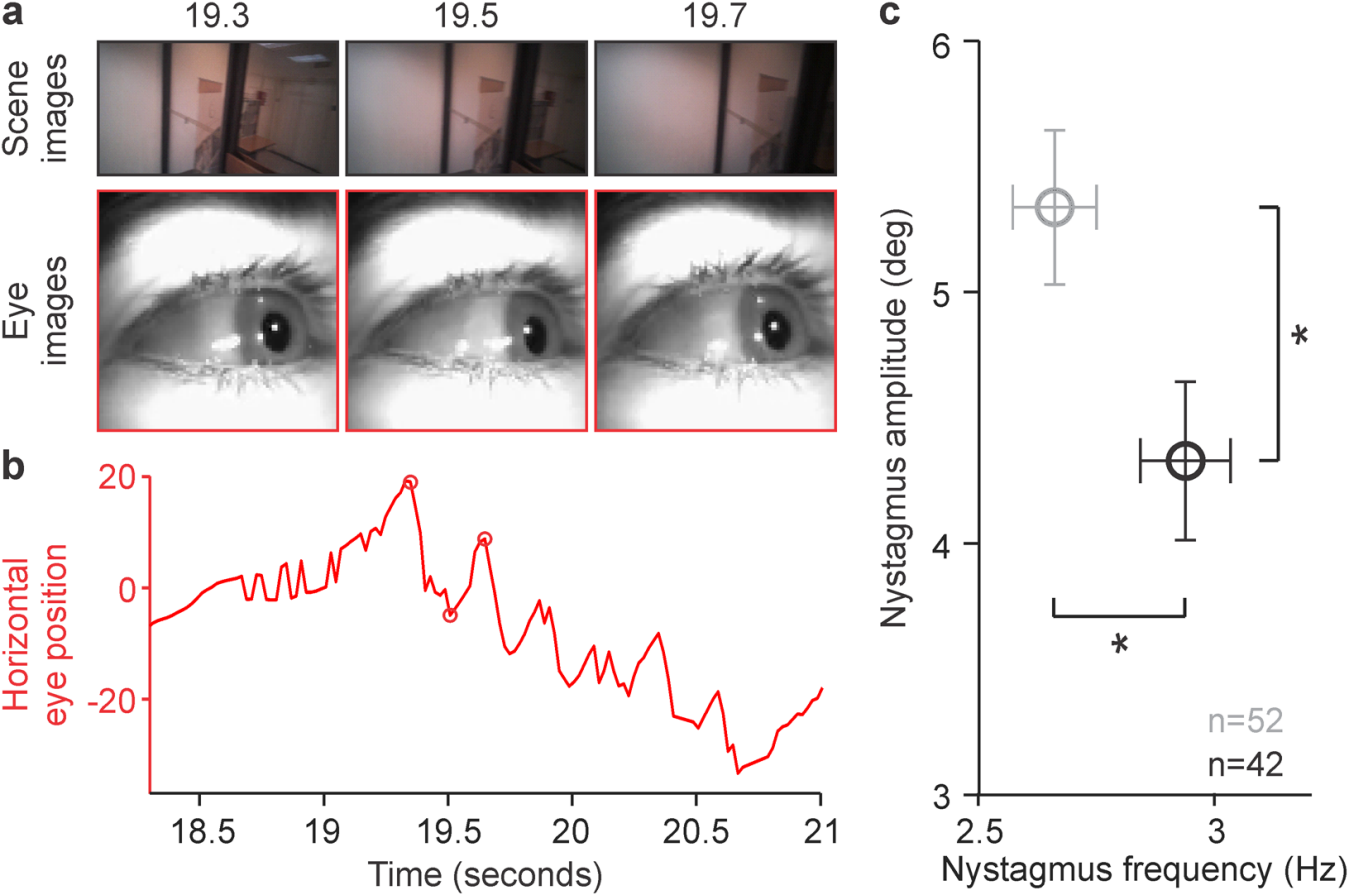
Turn-induced optokinetic nystagmus. (a) Scene (top) and eye images during a walking turn (bottom). Time in seconds at the top. (b) Eye horizontal position during the turn (circles: images in a). As in the optokinetic nystagmus induced with stripe patterns in stationary subjects, the eyes drift slowly before they start oscillating. Unlike in stationary subjects, the turn-induced reflex is briefer and more symmetric due to changes in stimulus acceleration and velocity. (c) The average turn-induced nystagmus had higher amplitude and lower frequency in five myopes (gray) than three emmetropes (black). Error bars: standard deviations. Asterisks: *p* = 0.01, Wilcoxon tests (*n*: number of nystagmus cycles).

Walking also triggered optokinetic reflexes when turning, as previously reported during turns at much faster speeds when driving cars or bicycles (Lappi et al., 2020; Vansteenkiste, Cardon, D'Hondt, Philippaerts, & Lenoir, 2013). As the subjects turned (Figure 9a), the eyes slowly drifted and then started oscillating at a frequency of 2–4 Hz (Figure 9b; see Movie 4). The turn-induced optokinetic reflex that we discovered is very different from the classical optokinetic reflex measured in stationary subjects. Unlike in the classical optokinetic reflex, the body, head, and eyes of our subjects were all moving and generating a complex pattern of scene motion that was continuously changing in both acceleration and velocity. The duration of the turn-induced optokinetic reflex was also brief and restricted to the time of the body turn. These stimulation differences made oscillations in eye position more symmetric in the turn-induced optokinetic reflex than in stationary subjects. Similarly, in head-fixed mice, eye position oscillations generated by optokinetic reflexes are more symmetric when induced with sinusoidally modulated stimulus velocity than constant velocity (Franca de Barros, Schenberg, Tagliabue, & Beraneck, 2020).

We investigated the properties of the turn-induced optokinetic reflex by measuring the average horizontal motion direction of the scene. As the subjects turned, the scene flow became strongly dominated by a horizontal direction that peaked in the middle of the turn. Most subjects had similar peaks at specific path locations (Supplementary Figure S10, asterisks, notice that the arrival time to the turn is not exactly the same across subjects), but the scene flow was different, reflecting the diversity of body–head movements, turning speeds, and head positions while turning. Subjects also differed in the number and strength of flow peaks, as well as the amplitude and frequency of lateral scene oscillations caused by swaying from left to right as they walked (Supplementary Figure S10a, b, lateral oscillation best visible in S1, S3, and S8). The turn-induced optokinetic reflexes also differed in amplitude and dominant frequency across subjects (Supplementary Figure S10c), and in the limited number of subjects that we tested, the amplitude of the reflex was significantly larger and the dominant frequency significantly lower in myopes than emmetropes (Figure 9c). It is important to emphasize that both turn-induced reflexes and reading generate oscillatory motion in the visual scene (Figure 1a, top panel). However, unlike the optokinetic reflex, reading makes the image oscillate from left to right in the absence of body movement and vestibular stimulation.

Optokinetic reflexes are known to be strongly dependent on the spatial frequency of the stimulus; therefore, we measured the average spatial frequency spectrum of the two tasks (Supplementary Figure S10d). As expected, walking generated images with significantly more power at low spatial frequencies than reading (3.83 ± 0.02 for walking vs. 3.71 ± 0.05 for reading, log_10_ power at 0.05 cycles per degree, *p* < 0.0001, Wilcoxon test), whereas reading generated images with significantly more power at high spatial frequencies than walking (−5.36 ± 0.13 for reading vs. −5.93 ± 0.04 for walking, log_10_ power at 20 cycles per degree, *p* < 0.0001, Wilcoxon test). Optokinetic reflexes are also strongly dominated by ON pathways in a large variety of species, including fish, rodents, lagomorphs, carnivores, primates, and humans (Emran et al., 2007; Sugita et al., 2013; Winkelman et al., 2019). Therefore, we conclude that, when compared with walking, reading reduces the stimulation of ON pathways driving optokinetic reflexes.

## Discussion

We have demonstrated that walking and reading stimulate ON visual pathways very differently. Whereas walking drives ON and OFF pathways with similar contrast across the retina, reading black letters on a white background drives the peripheral retina more strongly than the fovea, and within the fovea, it drives ON pathways less effectively than OFF pathways. When compared with walking, reading also decreases the stimulation of ON visual pathways through a reduction in retinal illumination, luminance contrast, luminance transients, and visual motion (Luo-Li et al., 2018; Mazade et al., 2019; Mazade et al., 2022; Rahimi-Nasrabadi et al., 2021; Winkelman et al., 2019; Xing et al., 2014).

### Reading reduces visual stimulation of ON pathways

Reading decreases retinal illumination by making the pupil smaller, which increases the depth of focus at short viewing distances (Feil, Moser, & Abegg, 2017; McDougal & Gamlin, 2015). Whereas the near-pupil reflex is absent in young children at elementary school (Schaeffel, Wilhelm, & Zrenner, 1993), it is present in older children who spend many hours reading at middle and high school. The reduction in retinal illumination through pupil constriction should become even more pronounced when reading indoors and can strongly reduce ON pathway activation (Mazade et al., 2019; Mazade et al., 2022; Rahimi-Nasrabadi et al., 2021) while increasing the risk of myopia progression (Ashby et al., 2009).

Reading also causes a dramatic reduction of the luminance transients driving strong responses from ON pathways. The reduction in luminance transients is mediated by both a decrease in blink frequency and scene change (i.e., the eyes are fixating on the same white-page scene for prolonged periods of time). Because ON pathways have more biphasic impulse responses than OFF pathways, ON pathways respond stronger than OFF pathways to luminance transients and weaker to stationary large surfaces (Jin, Wang, Lashgari, et al., 2011; Komban et al., 2014; Mazade et al., 2019; Mazade et al., 2022; Xing et al., 2014). Luminance transients also reduce myopia progression (Crewther & Crewther, 2002), and the duration of the dark period preceding a luminance transient is correlated with both the strength of ON pathway responses (Mazade et al., 2019) and the magnitude of myopia suppression (Schwahn & Schaeffel, 1997). Reading also eliminates visual motion and, by doing so, completely shuts down the stimulation of ON visual pathways driving optokinetic reflexes, which are crucial to eliminate motion blur and extremely well preserved through evolution (Dhande, Stafford, Lim, & Huberman, 2015; Dryja et al., 2005; Emran et al., 2007; Simpson, 1984; Sugita et al., 2013; Winkelman et al., 2019).

**Figure 10.**
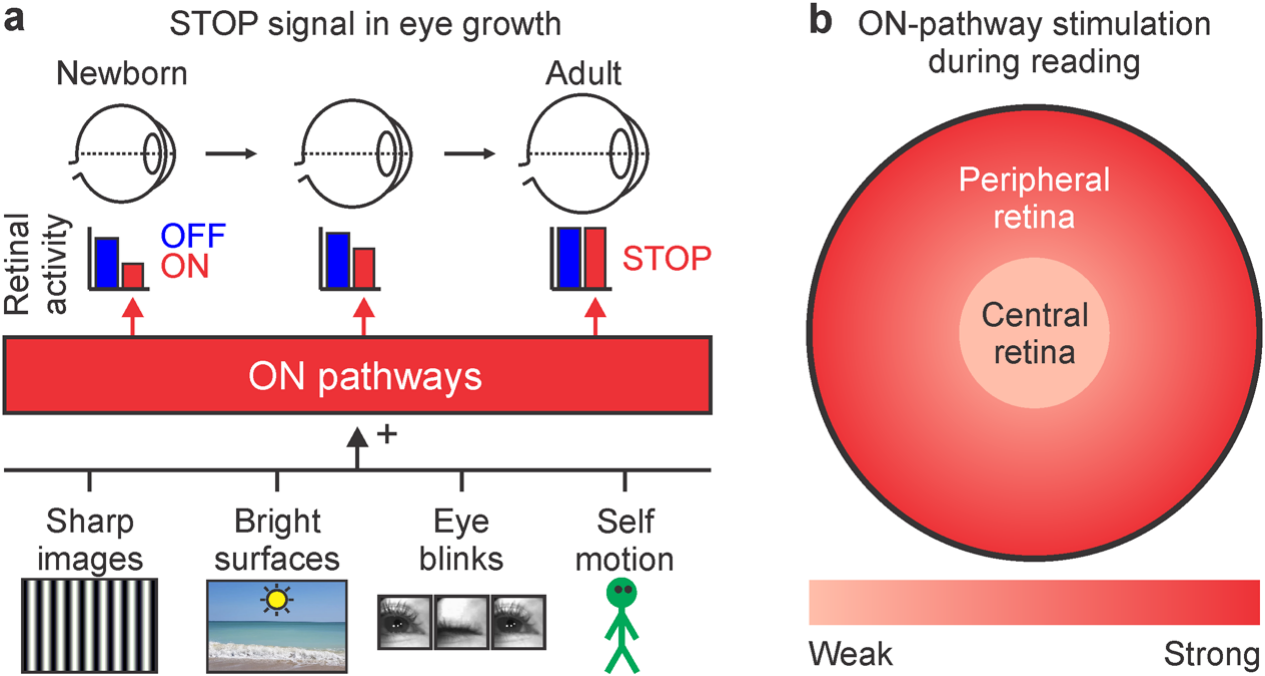
ON pathway activity as a stop signal for eye growth. (a) As the eye grows and its axial length approaches the focal length (top), retinal images become sharper, brighter, and more effective at stimulating ON visual pathways. ON visual pathways are strongly stimulated by high spatial frequencies, bright surfaces, eye blinks, and image stabilization circuits that are activated during visual navigation (bottom). Ideally, the eye should stop growing when the retinal response of the ON-pathway is as strong as for the OFF-pathway, a condition that requires retinal images to be sharpest, brightest, and change at the frequency that best activates ON pathways (middle). (b) Reading reduces ON pathway stimulation in the central retina more than in peripheral retina.

Walking also generates retinal images with a balanced content of light and dark contrast that should activate roughly equally ON and OFF visual pathways. Conversely, reading black text on a white background generates images heavily skewed toward dark contrast that decreases the stimulation of ON visual pathways. Therefore, reading weakens the visual stimulation of ON visual pathways through a reduction in retinal illumination, luminance transients (including eye blinks), self-motion, and light contrast (Figure 10a). The reduction in light contrast is most pronounced at the central retina, which is the retinal region most affected by myopia progression (Figure 10b).

### ON pathway activity provides an ideal stop signal for eye growth

The response properties of the ON pathway are ideal to signal when the retina is at the plane of focus. As the eye increases its axial length during development and approaches its focal distance, the population response from ON pathways should become increasingly stronger due to their preference for higher spatial frequencies than OFF pathways (Jansen et al., 2019; Kremkow et al., 2014; Pons et al., 2019; Pons et al., 2019). Consistently with this mechanism, visual responses are strongly dominated by OFF pathways early in development (Albus & Wolf, 1984; Wong & Oakley, 1996), and the OFF dominance becomes less pronounced in the adult brain (Jansen et al., 2019; Jin, Wang, Swadlow, & Alonso, 2011; Jin et al., 2008; Kremkow et al., 2014; Yeh, Xing, & Shapley, 2009).

Low spatial frequencies and dim light also weaken the visual responses from ON pathways (Jansen et al., 2019; Kremkow et al., 2014; Pons et al., 2019; Pons et al., 2017). Therefore, the population response of ON pathways should reach its maximum strength when the eye is at the focus plane and makes the retinal images sharpest and brightest (Figure 10a). The ON pathways driving the optokinetic reflex should also reach their maximum response when the retina is stimulated with the optimal combination of spatial frequency and velocity during visual navigation. The optimal spatial frequency and velocity should vary across species to match the receptive field sizes of retinal neurons and the spatiotemporal properties of the surrounding visual environment. For example, species that have a retinal fovea to search for targets at far distances (e.g., primates and birds of prey) may require high spatial frequencies moving at very slow velocities to optimally stimulate the small receptive fields of the fovea because targets at far distances are small and move slow. The need to detect slow motion also requires large eyes that may grow until the retina can detect the slowest object displacements in spatial position (e.g., a slow object movement of 1 degree can activate two separate retinal ganglion cells if separated by 300 microns in a big human eye but not if separated by 30 microns in a small mouse eye). Conversely, animals that do not require high visual acuity to search for food and that spend most of their time in small spaces and environments surrounded by shorter distances such as small caves (e.g., rodents) need lower spatial frequencies moving at faster velocities to optimally stimulate the retina. They can also have smaller eyes than animals that need to detect higher spatial frequencies and slower movements. Therefore, the stimulation of ON pathways should be maximized when the eye is at the plane of focus and exploring visual environments at the optimal viewing distance for each retina. Conversely, if the central retina is not properly stimulated during reading (Figure 10b), the eye should keep growing until it reaches the focal length that drives the maximum ON pathway activation.

The mechanism that we are proposing could also explain why positive and negative blur have opposite effects on eye growth (Schaeffel, Glasser, & Howland, 1988). Luminance transients drive stronger responses from OFF than ON pathways when the stimulus is small but stronger responses from ON pathways than OFF pathways when the stimulus is a large surface (Mazade et al., 2019; Mazade et al., 2022; Pons et al., 2019). Because stimuli are magnified by spectacles with positive blur and minified by spectacles with negative blur, positive and negative blur should have opposite effects in ON/OFF response balance. Negative lenses also blur near vision more than far vision, whereas positive lenses blur far vision more than near vision. Therefore, if the content of midrange spatial frequency driving eye growth (Flitcroft, Harb, & Wildsoet, 2020; Swiatczak & Schaeffel, 2021) is higher in near than far vision, negative lenses will cause a stronger reduction in midrange spatial frequencies than positive lenses. Midrange spatial frequencies are higher at near than far distances in natural environments (Torralba & Oliva, 2003), and the same may be true for the environments of animal models (e.g., rich textures of skin and fur from other animals at near distance have higher spatial frequencies than homogeneous walls at far distances). The human visual system also associates low spatial frequencies with far distances and high spatial frequencies with near distances, and this association is very strong. For example, when two gratings with different spatial frequencies are superimposed, the one with lower spatial frequency always appears to be farther away than the one with higher spatial frequency (Brown & Weisstein, 1988).

It is important to note that spatial frequencies below 10 cycles per degree are likely to play a more important role in driving myopia progression than higher spatial frequencies (Flitcroft et al., 2020; Swiatczak & Schaeffel, 2021; Tran, Chiu, Tian, & Wildsoet, 2008) simply because the retinal population response is strongest to spatial frequencies lower than 10 cycles per degree. Foveal retinal neurons respond strongly to these low frequencies because the spatial frequency bandwidth of retinal ganglion cells is broad (McMahon, Lankheet, Lennie, & Williams, 2000; Reinhard & Munch, 2021). Spatial frequencies lower than 10 cycles per degree are also better represented in visual scenes and generate stronger responses in human primary visual cortex than higher frequencies (Broderick, Simoncelli, & Winawer, 2022). Human readers are also most sensitive to spatial frequencies between 2 and 6 cycles per degree (Patching & Jordan, 2005). Therefore, whereas the resolution of human central vision is high enough to discriminate 60 cycles per degree, lower spatial frequencies (e.g., 2–6 cycles per degree) drive stronger responses in both retina and visual cortex. That being said, at each visual eccentricity and spatial frequency range, ON pathways respond stronger than OFF pathways to the higher end of the spatial frequency range, whereas OFF pathways respond stronger than ON pathways to the lower end. Therefore, by reducing the power of high spatial frequencies, optical blur should affect the responses to grating patterns and edges in ON more than OFF pathways.

The notion that ON/OFF response balance plays an important role in eye growth is supported by an increasingly larger number of studies (Aleman et al., 2018; Chakraborty et al., 2015; Crewther & Crewther, 2002; Crewther & Crewther, 2003; Iuvone et al., 1991; Pardue et al., 2008; Pons et al., 2019; Pons et al., 2017; Schwahn & Schaeffel, 1997; Smith et al., 1991). First, the pharmacological inactivation of ON visual pathways affects ocular growth in both kittens and chickens (Crewther & Crewther, 2003; Smith et al., 1991). Second, mice without functional ON pathways become more susceptible to developing myopia (Chakraborty et al., 2015; Pardue et al., 2008), and humans with complete ON pathway deficits develop high myopia (Dryja et al., 2005; Kim et al., 2021). Third, injections of apomorphine, a dopamine receptor agonist that stimulates ON dopaminergic amacrine cells, prevent myopia progression in rhesus monkeys (Iuvone et al., 1991). Fourth, luminance transients that drive strong responses from ON visual pathways (Mazade et al., 2019; Mazade et al., 2022) reduce myopia progression in chickens (Crewther & Crewther, 2002). And fifth, increasing the dark periods preceding luminance transients strengthens the ON pathway responses (Mazade et al., 2019) and suppresses myopia progression more effectively (Schwahn & Schaeffel, 1997).

### Diversity of ON visual pathways

ON visual pathways are very diverse in their anatomy and function. They originate in at least seven different types of bipolar cells in primates (Tsukamoto & Omi, 2016) and more than 10 different types of retinal ganglion cells in mice (Baden et al., 2016), and they project to multiple brain structures, including the lateral and ventral geniculate nucleus, superior colliculus, and accessory optic system (Dhande et al., 2015). And yet, all ON visual pathways share important properties in common that make them more vulnerable than OFF pathways to loss of image brightness, contrast, sharpness, and motion. All ON visual pathways are driven by the onset of light stimuli and need slow metabotropic glutamate receptors to invert the hyperpolarizing currents from the photoreceptors (Masu et al., 1995; Slaughter & Miller, 1981). These properties make ON pathways dependent on bright stimuli and slow integration times that may help to process slow motion (Luo-Li et al., 2018). As a group, ON pathways also have higher contrast sensitivity and contrast–response saturation than OFF pathways (Chichilnisky & Kalmar, 2002; Kremkow et al., 2014; Rahimi-Nasrabadi et al., 2021; Rahimi-Nasrabadi et al., 2022; Soto et al., 2020; Zaghloul et al., 2003), a difference that is likely to originate at the photoreceptor (Kremkow et al., 2014; Lee, Dacey, Smith, & Pokorny, 2003). The higher contrast sensitivity of ON than OFF pathways maximizes the sampling efficiency of light and dark contrast in our visual world (Rahimi-Nasrabadi et al., 2021) but also makes ON pathways more vulnerable to optical blur than OFF pathways (Kremkow et al., 2014; Pons et al., 2019; Pons et al., 2017).

### The role of visual experience in myopia progression

Many studies have demonstrated that reading increases the risk of developing myopia (Kinge, Midelfart, Jacobsen, & Rystad, 2000; Mirshahi et al., 2014; Morgan & Rose, 2005; Morgan et al., 2018; Morgan & Jan, 2022; Mutti et al., 2002; Mutti & Zadnik, 1995; Pärssinen & Lyyra, 1993; Rozema et al., 2021; Saw, Hong, Chia, Stone, & Tan, 2001; Saw, Hong, et al., 2001; Zylbermann et al., 1993), and a similar effect has been demonstrated in other tasks that require fixating the eye at near distances such as in the quality control of textiles (Simensen & Thorud, 1994). Opposite to reading, spending time outdoors reduces the risk of myopia progression (French, Ashby, Morgan, & Rose, 2013; Sherwin et al., 2012; Xiong et al., 2017), and myopia prevalence can be nearly an order of magnitude lower in rural India than urban Singapore, presumably because of differences in outdoor activity (Morgan & Rose, 2005).

Whereas visual stimulation plays an important role in myopia, the specific stimulus parameters driving myopia progression remain poorly understood. Monocular deprivation causes high myopia in animal models (Raviola & Wiesel, 1985) through a reduction in retinal dopamine (Stone, Lin, Laties, & Iuvone, 1989; Zhou, Pardue, Iuvone, & Qu, 2017), which is released by dopaminergic amacrine neurons driven by the ON pathway (Munteanu et al., 2018). Optical blur induced with negative lenses also causes myopia in animal models (Hung, Crawford, & Smith, 1995; Schaeffel et al., 1988) through mechanisms that remain poorly understood but are thought to involve glucagon-containing amacrine cells in chicks (Fischer, McGuire, Schaeffel, & Stell, 1999). Whereas light intensity and optical blur are important factors in myopia development, we still do not understand why reading increases myopia progression and outdoor activity reduces it. Without a more detailed understanding of the stimulation parameters driving myopia, it is difficult to develop effective programs of myopia prevention, and school program reforms solely based on increasing outdoor activity had limited success (He et al., 2015; Jonas et al., 2021; Morgan & Jan, 2022; Wu et al., 2020; Xiong et al., 2017). The mechanism that we propose predicts that preventing myopia progression will require a visual diet that includes high retinal illuminance, high contrast, frequent luminance transients, and enough visual motion to drive reflexes of image-retinal stabilization. Reading white letters on a black background increases the light contrast driving ON pathways (Aleman et al., 2018) but may not be enough to prevent myopia progression because it reduces retinal illumination and lacks visual motion. Therefore, our results indicate that the best approach to control myopia progression is spending *active* time outdoors, which requires engaging children in tasks involving visual navigation.

## Supplementary Material

Supplement 1

Supplement 2

Supplement 3

Supplement 4

Supplement 5
