# Supplementary material for: Differences in visual stimulation between reading and walking and implications for myopia development
Source: J Vis. 2023 Apr 4;23(4):3. doi: 10.1167/jov.23.4.3 (PMC10080958; doi:10.1167/jov.23.4.3)
Supplement: Supplement 1 [file jovi-23-4-3_s001.pdf]

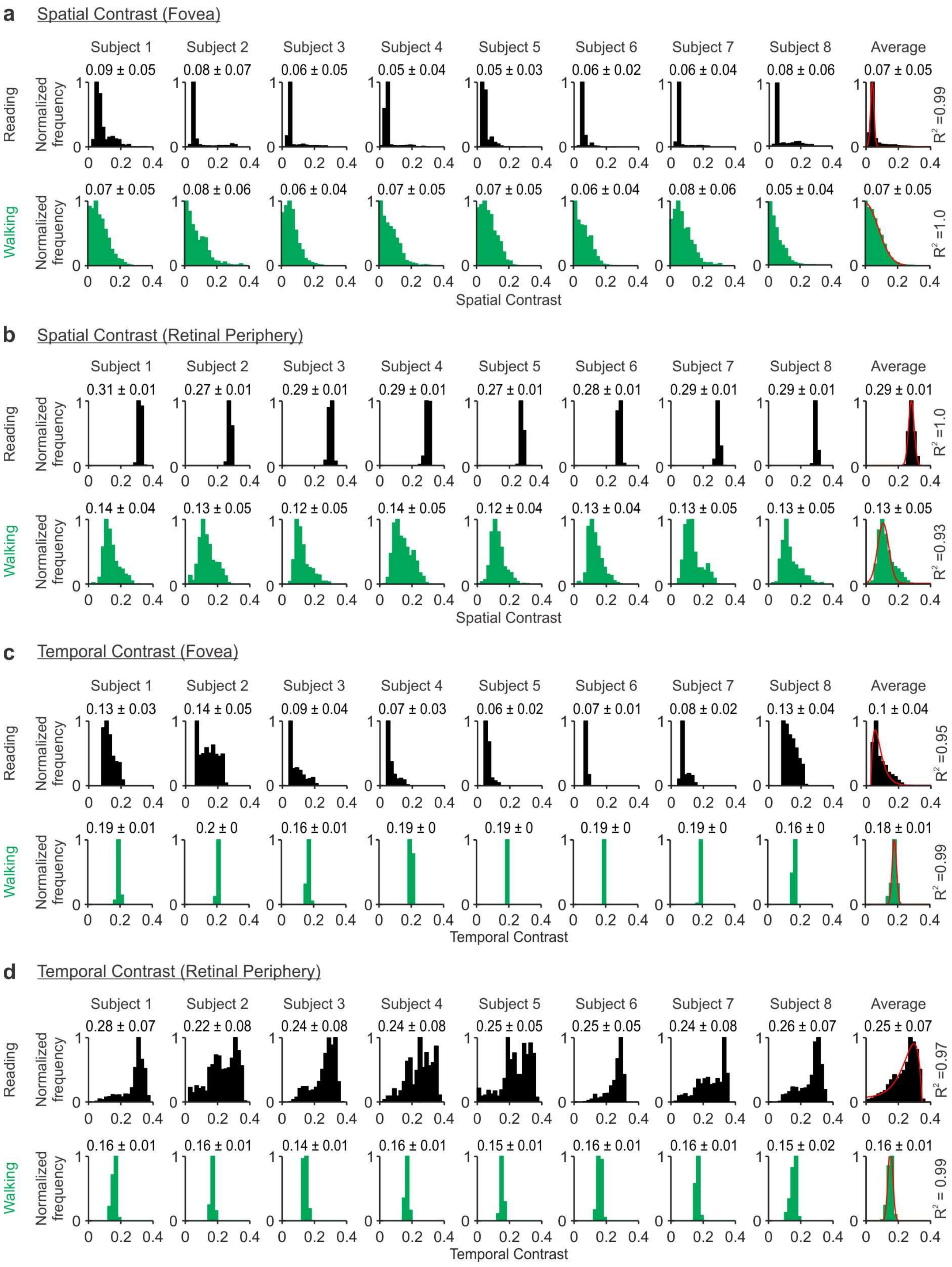

Supplementary Figure 1: Distributions of image spatial and temporal contrast. a. Histograms of spatial contrast measured at the fovea of eight subjects (1-8) and the average across subjects (right panel) in reading task (black, top row) and walking task (green, bottom row). The numbers on the top of each histogram represent means ± standard deviations of spatial contrasts, which range from 0 to 1. The red lines superimposed on the average histograms are Gaussian fits (goodness of fit on the right). **b.** Same as a, for visual periphery. **c.** Same as a, for temporal contrast. The red lines are Alpha fits for reading and Gaussian fits for walking. **d.** Same as b for temporal contrast.

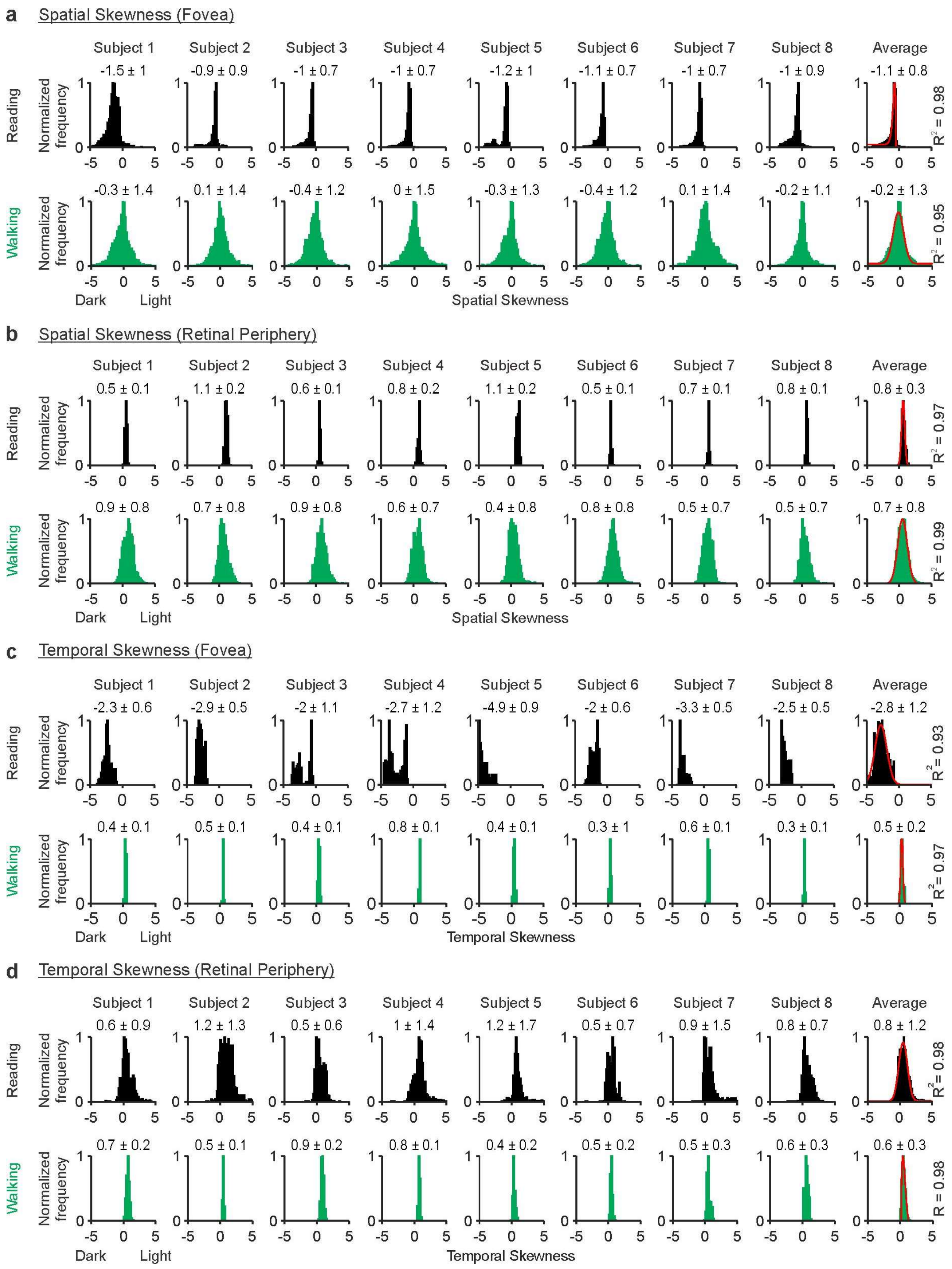

Supplementary Figure 2: Distributions of image spatial and temporal skewness. a. Histograms of spatial skewness measured at the fovea of eight subjects (1-8) and the average across subjects (right panel) in reading task (black, top row) and walking task (green, bottom row). The format is the same as in Figure 2. **b.** Same as a, for visual periphery. **c.** Same as a, for temporal skewness. **d.** Same as b, for temporal skewness.

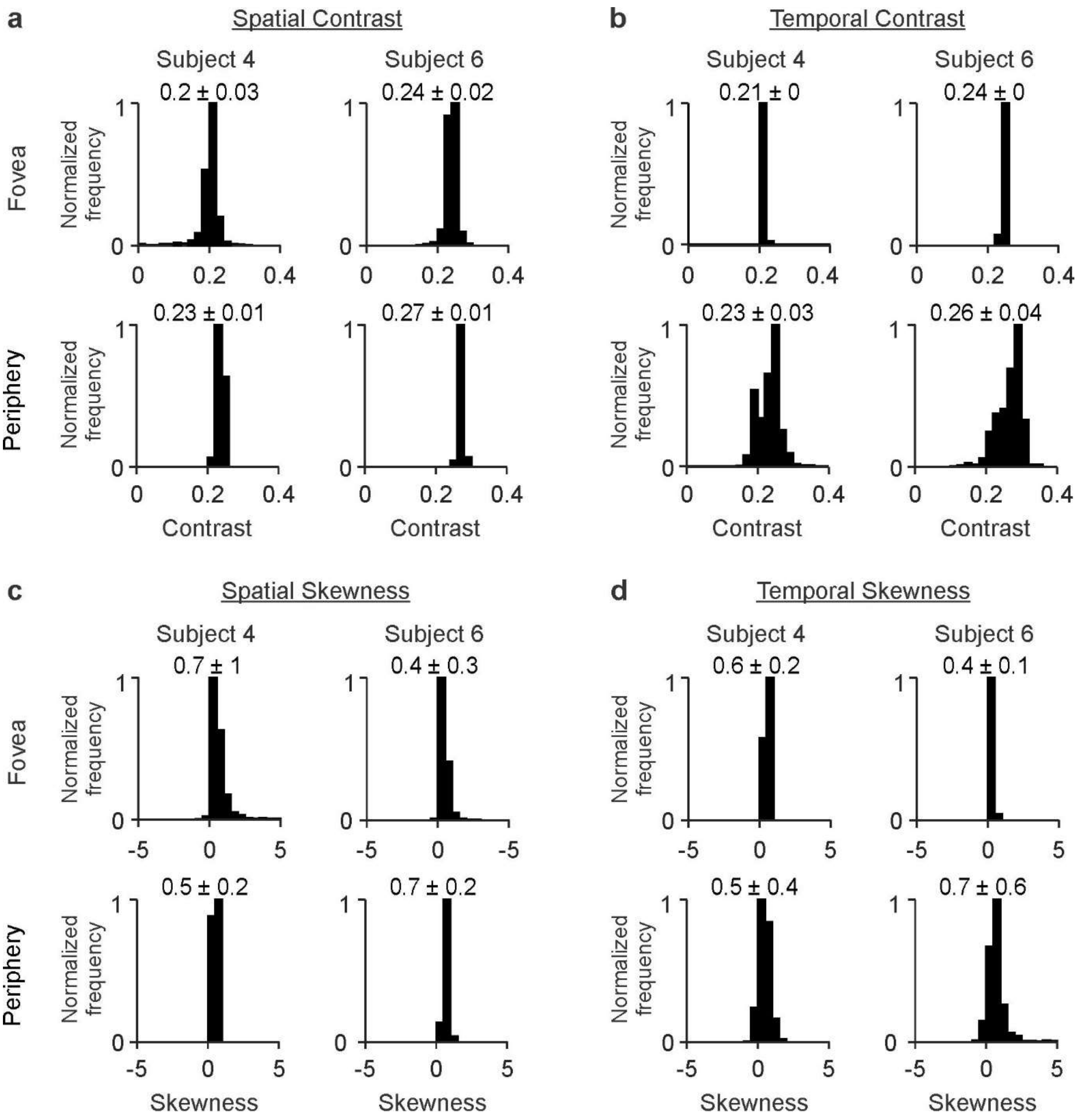

Supplementary Figure 3: Reading white text on black background increases the balance of light and dark contrasts. a. Top**:** RMS spatial contrast measured at the fovea of two example subjects (left: sub 4, right: sub 6) when reading white text on black background. Bottom: Same as a for visual periphery. **b.** Same as a, for RMS temporal contrast. **c.** Same as a, for spatial skewness. **d.** Same as a, for temporal skewness.

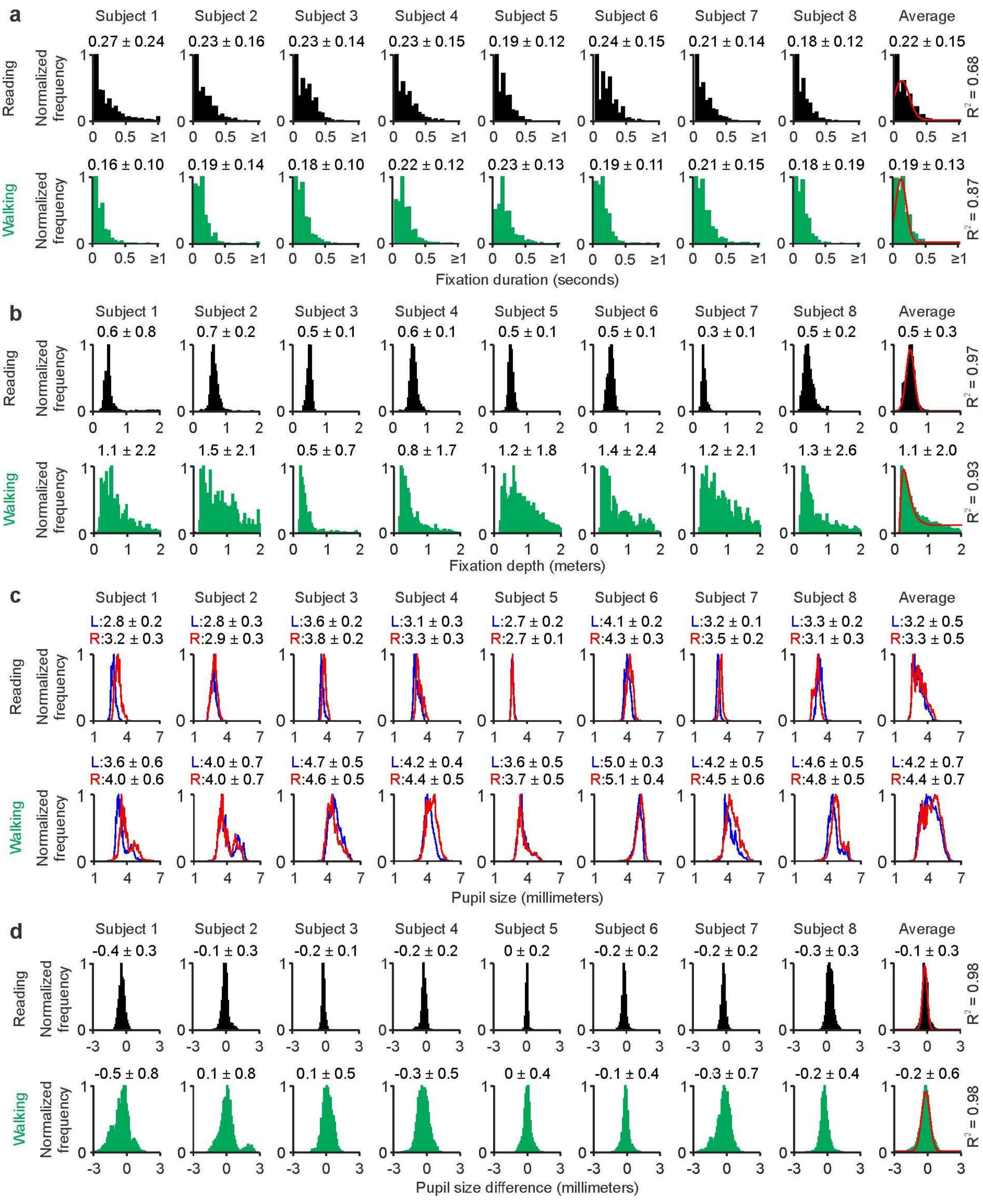

Supplementary Figure 4: Distributions of fixation dynamics and pupil size. a. Histograms of fixation duration measured in eight subjects (1-8) and the average across subjects (right panel) in the reading task (black, top row) and walking task (green, bottom row). The format is the same as in Figure S1. **b.** Same as a, for fixation distance. **c.** Line plots of left (blue) and right (red) pupil sizes. **d.** Same as a, for pupil size differences (left-right pupil sizes).

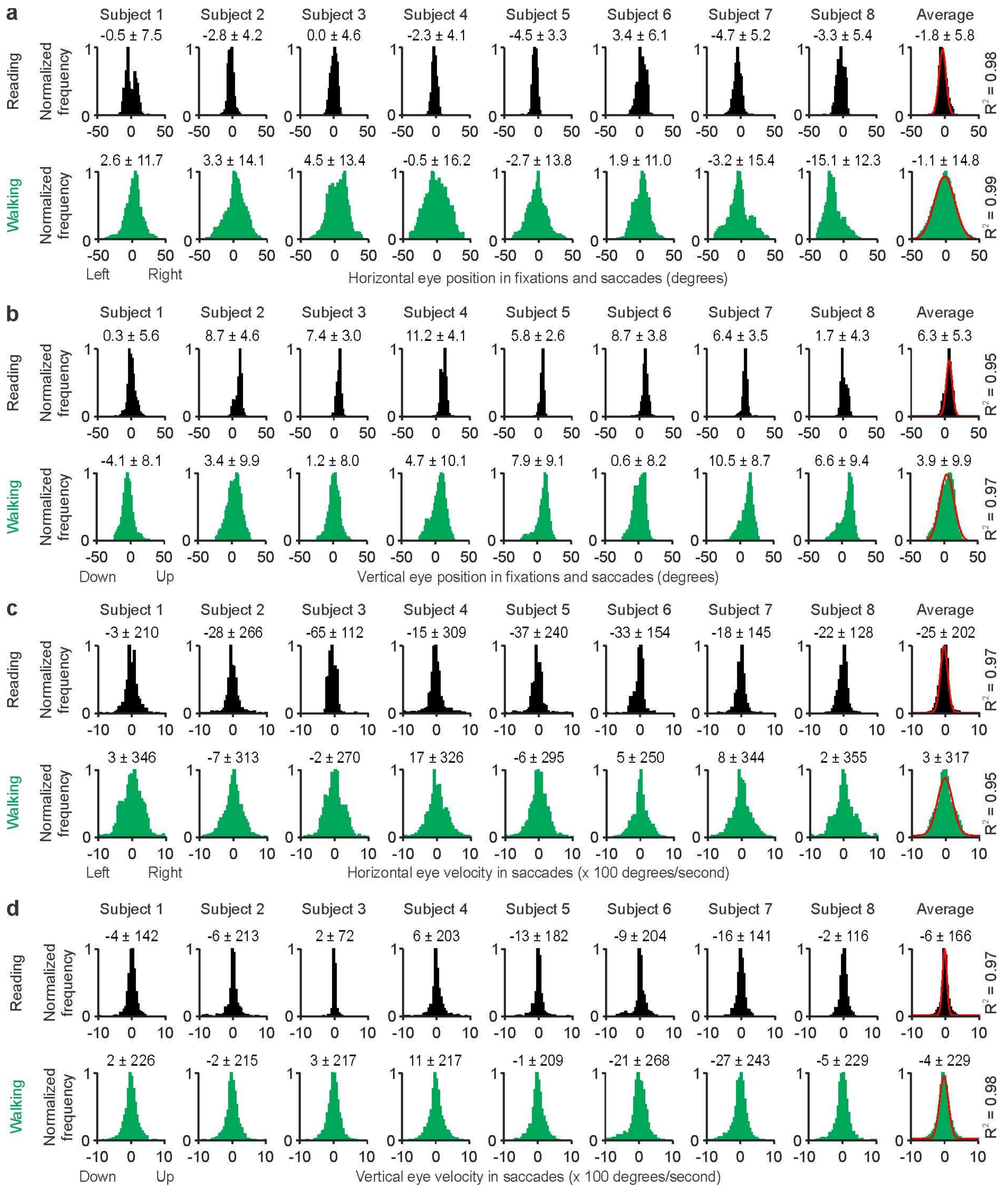

Supplementary Figure 5: Distributions for eye movements. a. Histograms of horizontal eye movements measured in eight subjects (1-8) and the average across subjects (right panel) in the reading task (black, top row) and walking task (green, bottom row). Eye movements include both fixations and saccades. The format is the same as in Figure S1. The [means, standard deviations] of the Gaussian fits are [-3.5, 5.3] for reading and [-1.6, 15.1] for walking. **b.** Same as a for vertical eye movements. The [means, standard deviations] of the Gaussian fits are [6.3, 4.5] for reading and [4.1, 10.6] for walking. **c.** Same as a for horizontal eye velocity (only for saccades). The [means, standard deviations] of the Gaussian fits are [-44.9, 109.9] for reading and [-23.0, 224.0] for walking. **d.** Same as a for vertical eye velocity. The [means, standard deviations] of the Gaussian fits are [-25.1, 64.7] for reading and [-19.8, 132.0] for walking.

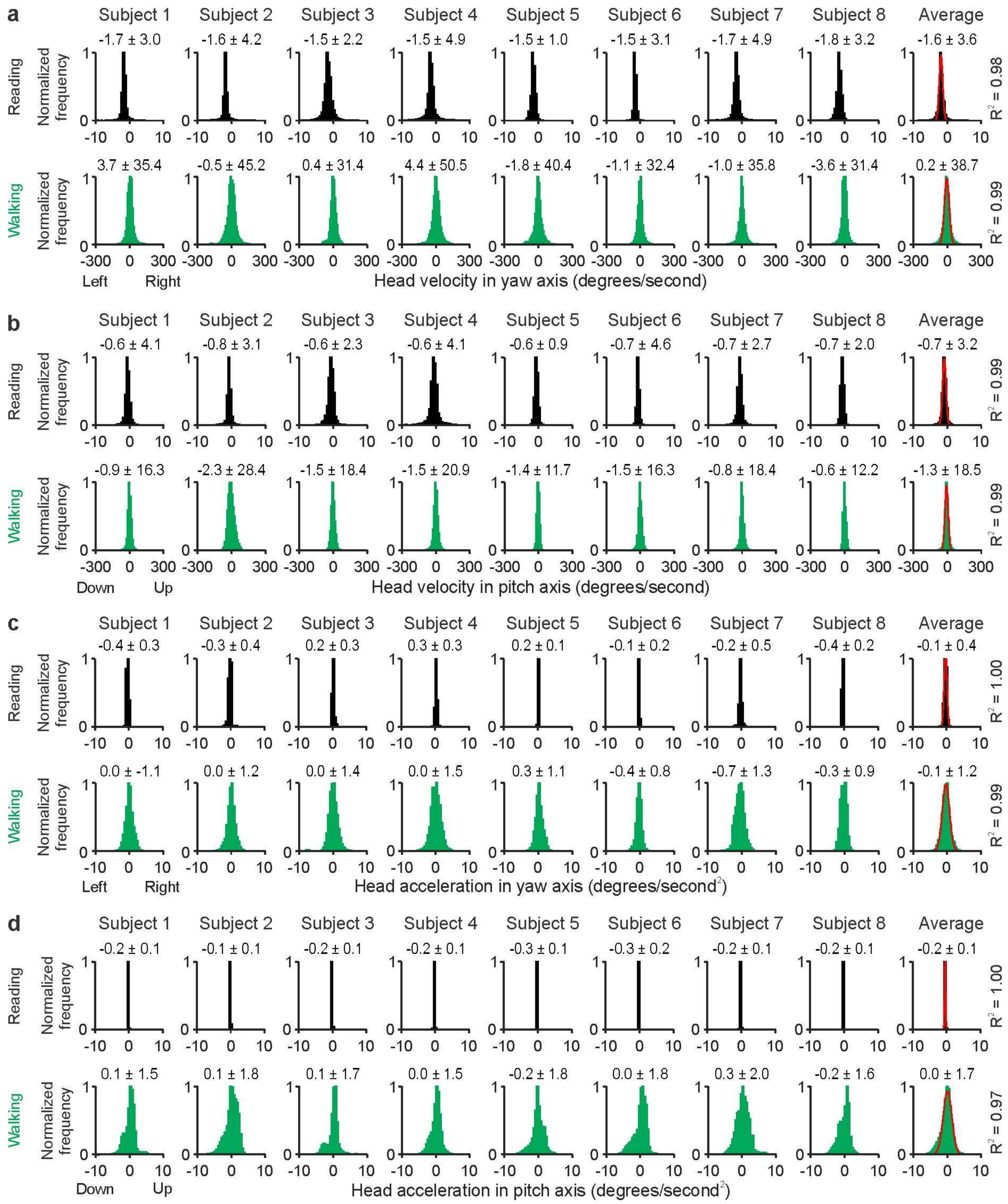

Supplementary Figure 6: Distributions for head motion. a. Histograms of head yaw velocity measured in eight subjects (1-8) and the average across subjects (right panel) in the reading task (black, top row) and walking task (green, bottom row). The format is the same as in Figure 2. The [means, standard deviations] of the Gaussian fits are [-1.9, 0.5] for reading and [-4.5, 21.2] for walking. **b.** Same as a for head pitch velocity. The [means, standard deviations] of the Gaussian fits are [-0.9, 0.5] for reading and [-7.3, 14.1] for walking. **c.** Same as a, for head yaw acceleration. The [means, standard deviations] of the Gaussian fits are [-0.3, 0.4] for reading and [-0.4, 1.1] for walking. **d.** Same as a, for head pitch acceleration. The [means, standard deviations] of the Gaussian fits are [-0.5, 0.2] for reading and [0.1, 1.3] for walking.

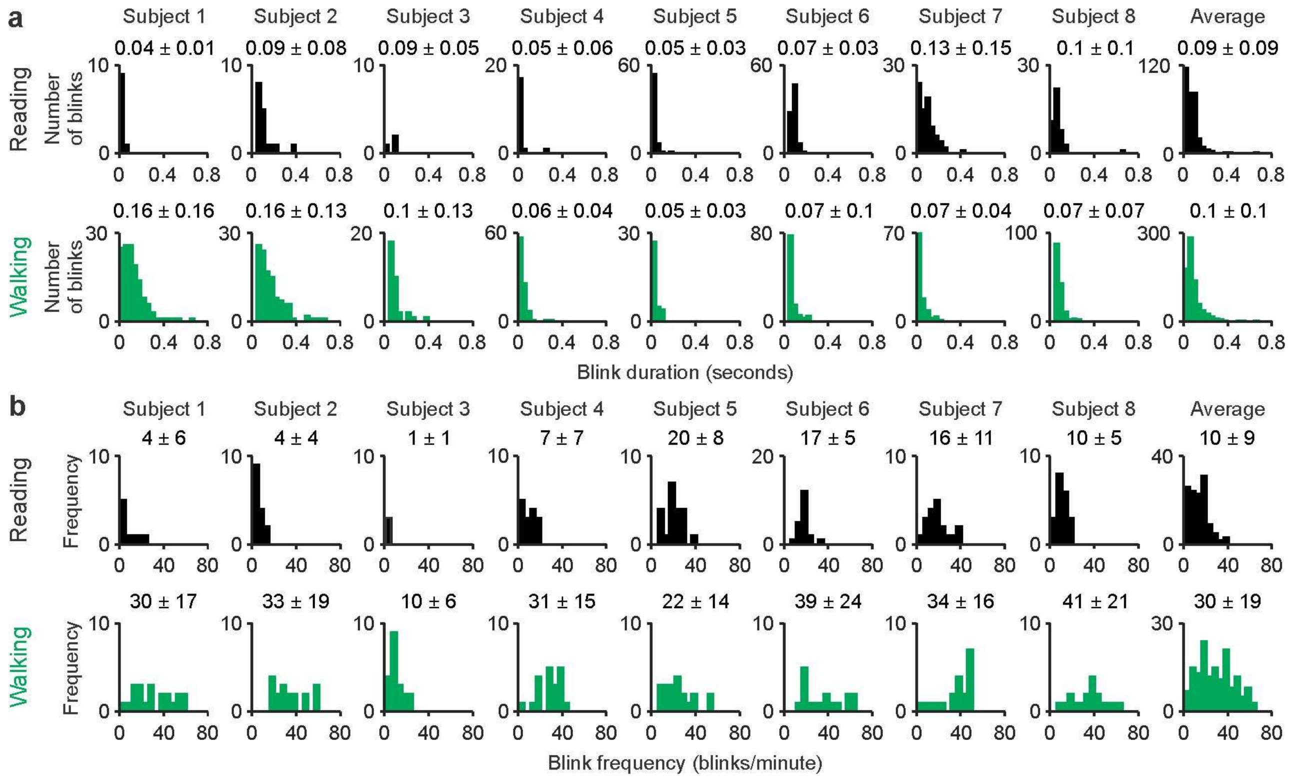

Supplementary Figure 7: Distribution for eye blinks. a. Histograms of blink duration measured in eight subjects (1-8) and the average across subjects (right panel) in the reading task (black, top row) and walking task (green, bottom row). The format is the same as in Figure S1. **b.** Same as a, for blink frequency.

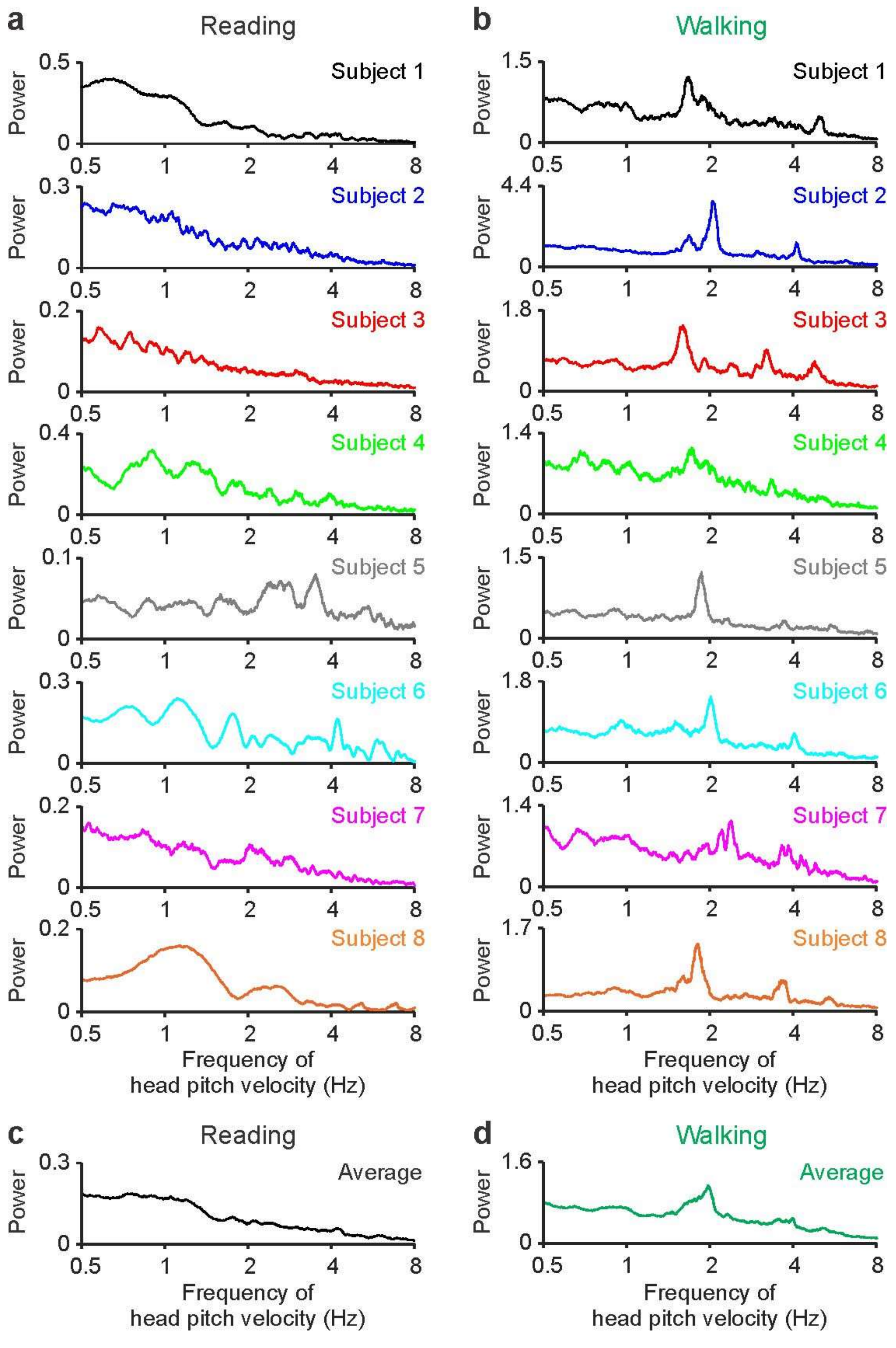

Supplementary Figure 8: Power spectrum of head pitch velocity. a. Power spectrum of head pitch velocity for eight subjects in the reading task. **b.** Same as a, in the walking task. **c.** Average power spectrum across subjects in the reading task. **d.** Same as c, in the walking task.

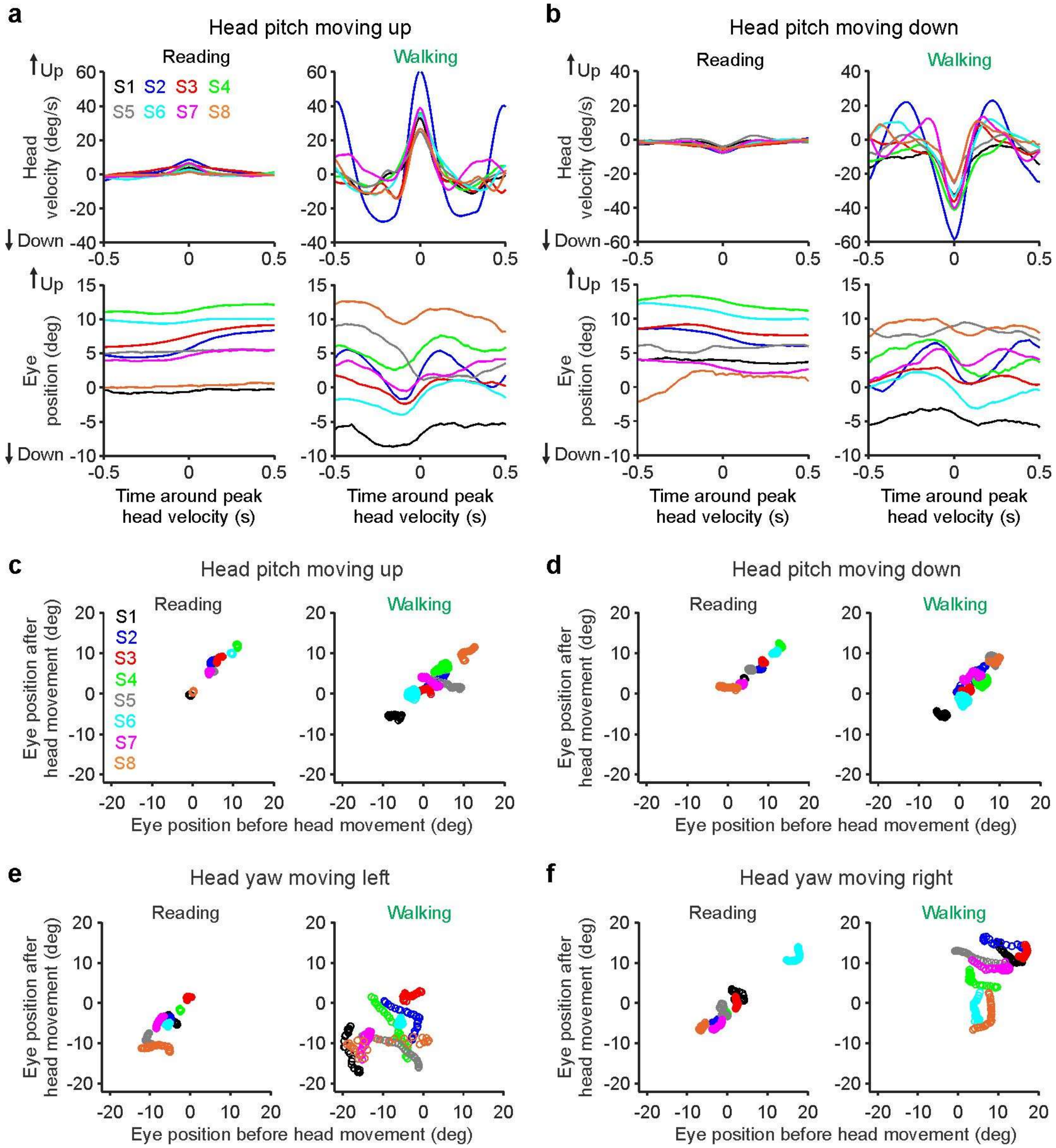

Supplementary Figure 9: Head-triggered eye movements. a. Top. Average velocity of head pitch moving up, centered at the point of maximum velocity for reading (top left) and walking (top right). **Bottom.** Average eye position centered at the point of maximum head velocity for reading (bottom left) and walking (bottom right). **b.** Same as a, for head pitch moving down. **c.** Scatter plot showing eye position before and after the maximum velocity of head pitch moving up, for reading (left) and walking (right). The data scatter could be accurately fit with linear functions (R^2: 0.95/0.93, slopes: 1.0/0.8, intercepts 0.9/0.7 for reading/walking). **d.** Same as c for head pitch moving down (R^2: 0.75/0.85, slopes: 0.7/1.1, intercepts 1.1/-0.9 for reading/walking). The average eye position showed slight variations across individual subjects. In most subjects, it was above the middle of the orbit in both tasks and remained remarkably stable as the head moved up and down. Across subjects, the average eye positions around a head movement could be accurately fit with a linear function with a slope close to one. **e.** Same as c, for head yaw moving left (R^2: 0.67, slope: 0.9, intercept: 0.4 for reading). **f.** Same as c, for head yaw moving right (R^2: 0.94, slope: 0.8, intercept: -2.0 for reading). Compared to head pitch movements, the average eye positions associated with head yaw movements were more variable and were better fit with a linear function during reading than walking. In both tasks, when the head moved towards the left, the average eye position was on the left side before and after the head movement. Similarly, when the head moved towards the right, the average eye position was on the right side before and after a head movement. During walking, changes in average eye position around yaw head movements followed a linear function with a negative slope in some subjects (e.g. subjects 1, 2, 4 and 5 in e-f walking panels). These negative slopes indicate that head movements towards the left made the eyes move towards the left if they were already centered at the orbit but towards the center of the orbit if they were already on the left side. Similarly, head movements towards the right made the eyes move towards the right if they were already centered at the orbit but towards the center of the orbit if they were already viewing the right side. Therefore, during walking, turning the head towards one side makes the eye move towards the orbit center when they eye is eccentrically viewing, but towards eccentric viewing when the eye is at the center of the orbit.

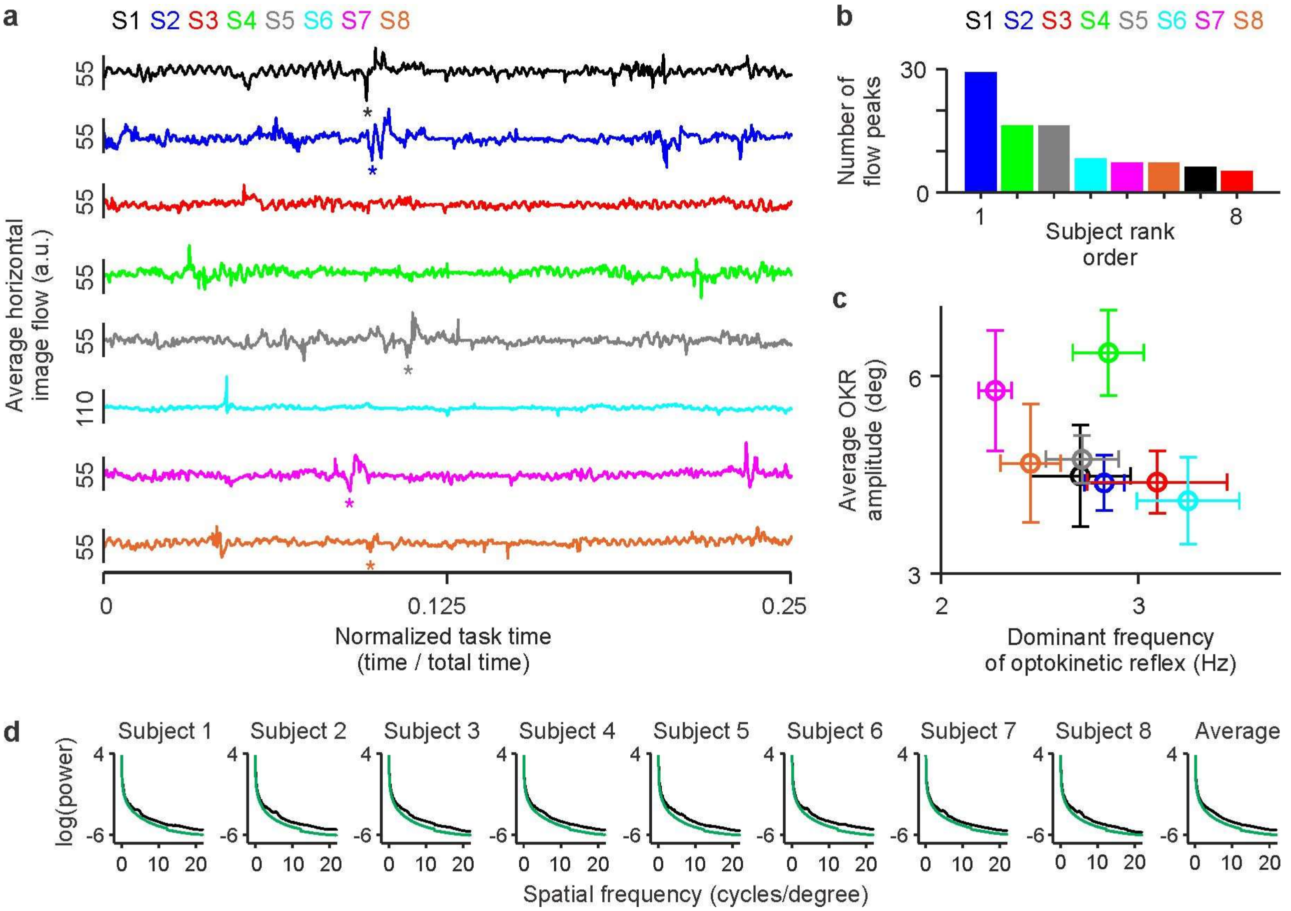

Supplementary Figure 10: Self-motion-induced optokinetic reflex. a. Average horizontal flow measured in eight subjects. Notice the similarity in the time of flow spikes (asterisks) when the subjects turn in the corridor. **b.** Bar plots showing the number of spikes in horizontal flow measured in eight subjects. **c.** Average amplitude and frequency of the optokinetic reflex for each subject. **d.** Power spectrum of scene image spatial frequency of eight subjects (1-8) and the average across subjects (right) for the reading task (black) and walking task (green).
